# Comparative study on the molecular mechanisms of *Trichoderma*, *Cladosporium* and *Penicillium* strains (Ascomycota) in positively regulating *Festuca sinensis* cv. Qinghai based on multi-omics

**DOI:** 10.3389/fmicb.2026.1788491

**Published:** 2026-03-25

**Authors:** Deyu Dong, Yuting Guo, Zhanling Xie, Xueting Fa, Yuan Gao, Baojie Deng, Jianing Yu

**Affiliations:** College of Ecological and Environment Engineering, Qinghai University, Xining, Qinghai, China

**Keywords:** carbon cycling, endophytic Ascomycota, *Festuca sinensis*, hierarchical gradient, hormone signal transduction, steroid biosynthesis

## Abstract

**Introduction:**

*Festuca sinensis* cv. Qinghai is a key ecological forage on the Qinghai-Tibet Plateau, but its slow natural growth hinders grassland restoration. Strains of the genera *Trichoderma*, *Cladosporium* and *Penicillium* are research hotspots in agriculture owing to their prominent plant growth-promoting and stress-tolerance enhancing properties. However, the differences in growth-promoting effects and underlying molecular mechanisms among their genera remain poorly elucidated.

**Methods:**

In this study, *F. sinensis* was treated with strains of endophytic Ascomycota (classified into the genera *Trichoderma*, *Cladosporium*, and *Penicillium*), followed by comprehensive analysis of forage growth-related traits and rhizospheric soil enzyme activities, combined with integrative metabolomics and transcriptomics.

**Results:**

The results demonstrated that treatment with endophytic fungi significantly increased the aboveground dry weight, root length, and tillering capacity of *F. sinensis*, and Cp78, Pe167, and Ta121 were selected for omics analysis due to their strongest growth-promoting effects, with a distinct hierarchical gradient in the complexity of growth-promoting mechanisms among the three genera: *Trichoderma* > *Cladosporium* > *Penicillium*. Further multi-omics analysis revealed divergent core mechanisms of the three genera. First, *Trichoderma* promoted growth via tryptophan metabolism (IAA synthesis), upregulation of hormone signal transduction genes/transcription factors, and enhanced biosynthesis of anti-disease metabolites (e.g., alkaloids). Second, *Cladosporium* activated the steroid biosynthesis pathway to produce brassinosteroid precursor substances, and promoted intracellular hormone transport and signal transduction by overexpressing signal transduction-related genes. Third, *Penicillium* primarily promoted plant growth by regulating key pathways involved in carbon cycling.

**Discussion:**

This study clarifies the genus-specific growth-promoting mechanisms of Ascomycete endophytes on *F. sinensis*, provides theoretical support and high-quality strain resources for developing microbial inoculants specific to alpine forages, and facilitates grassland restoration, with significant ecological and agricultural implications for alpine pastoral regions.

## 1 Introduction

In recent years, fungal inoculants have attracted increasing attention for their application value in agricultural production due to their environmentally friendly characteristics and remarkable ecological benefits (Tripathi et al., 2020). Currently, the most commonly used fungal inoculants mainly include *Trichoderma*, *Penicillium*, and *Cladosporium*, all of which belong to the Ascomycota (Boro et al., 2022; Feng et al., 2025; Ifunanya et al., 2023). Ascomycete inoculants can increase the content of soil mineral nutrients and regulate plant physiological metabolic processes to promote plant growth and development, as well as improve yield and quality. Meanwhile, they can also create a stable and suitable rhizosphere microecological environment for plant growth by regulating the structure of rhizosphere microbial communities, enhancing interactions among beneficial microorganisms, and inhibiting the reproduction of harmful microorganisms (Adenike et al., 2023; Mohamed, 2021; Rifat et al., 2010; Sharma et al., 2023). However, most studies have focused on the growth-promoting effects of ascomycetes, and comparative studies on the growth-promoting effects among different genera of ascomycetes remain scarce.

*F. sinensis* is an excellent ecological forage grass on the Qinghai-Tibet Plateau, characterized by its strong resilience to environmental stresses and rich nutritional content. In recent years, this species has played a crucial role in the ecological management of high-altitude pastoral areas on the plateau. Nevertheless, its limited seed availability leads to slow natural growth, which hinders the progress of grassland vegetation restoration on the Qinghai-Tibet Plateau. A large body of research has demonstrated that the symbiotic association between endophytes and their host forages is a stable mutualistic relationship (Wang and Dai, 2011). Consequently, the strategy of isolating beneficial endophytes from plants and developing them into microbial preparations has garnered significant attention. However, there is limited literature on beneficial endophytes isolated from alpine and cold regions and their application in promoting the growth of *F. sinensis*.

*Trichoderma* inoculants are particularly widely used due to their comprehensive functions and strong adaptability. *Trichoderma* strains can directly promote plant growth and development by secreting plant hormones and various bioactive substances (Khan et al., 2023). Meanwhile, they can inhibit the growth of pathogens by producing antagonistic substances or induce systemic disease resistance in plants, thereby enhancing plant defense against diseases (Feng et al., 2025; Yao et al., 2023). For instance, *Trichoderma guizhouense* can antagonize 74 species of plant pathogenic fungi through nutrient competition and hydrolase secretion, and further promote plant growth and development by producing bioactive compounds and colonizing plant roots (Xie et al., 2025). Similarly, a study conducted by Dong et al. (2025a,b) confirmed that seed soaking with *Trichoderma* suspension significantly improves the biomass accumulation of forages in the Qinghai-Tibet Plateau region (Dong et al., 2025b). *Trichoderma* strains with both growth-promoting and disease-resistant functions have broad application prospects in agricultural production (Guzmán-Guzmán et al., 2023; Waleed et al., 2024). Up to now, more than 50 commercial *Trichoderma* inoculants have been marketed worldwide, which further confirms their market recognition and application value (Lian et al., 2023). It should be noted, however, that some *Trichoderma* species can cause opportunistic infections in plants under specific environmental conditions or when host immunity is compromised, highlighting the need for rigorous strain selection for agricultural applications.

*Cladosporium* has received extensive attention and application in agricultural production and ecological restoration due to its diverse biological activities and beneficial interaction effects with plants (Yang et al., 2023). Numerous studies have verified that *Cladosporium* colonizing the plant rhizosphere can exert positive effects through multiple pathways: it can not only directly promote plant growth and development, stimulate root morphogenesis (e.g., increasing lateral root number and extending root length), and improve nutrient use efficiency, but also enhance plant tolerance to abiotic stresses such as drought and salt stress. Meanwhile, it can achieve biocontrol functions by inhibiting the proliferation of pathogenic microorganisms, thereby improving soil physicochemical properties and fertility levels (Hamayun et al., 2009a; Hamayun et al., 2009b; Raut et al., 2021). In addition, *Cladosporium* also contributes to regulating the diversity of soil microbial communities and improving environmental stability, playing an indispensable role in maintaining the health and balance of soil ecosystems (Long et al., 2026). Nevertheless, many *Cladosporium* species are known as plant pathogens that cause leaf spot diseases. In addition, some strains can act as opportunistic human pathogens, such as inducing allergic reactions or mycoses in immunocompromised individuals, underscoring the importance of evaluating the safety of candidate strains.

*Penicillium* plays an irreplaceable role in processes such as soil organic matter decomposition and fertility maintenance. *Penicillium* possesses high-efficiency rhizosphere colonization ability and is one of the important groups of plant growth-promoting fungi. Its growth-promoting effects on plants have been widely validated in various species including rice, wheat, and forages. Studies have shown that after colonizing the plant rhizosphere, *Penicillium* can not only directly promote plant growth and development and improve nutrient absorption efficiency, but also enhance plant tolerance to abiotic and biotic stresses such as drought, salt stress, diseases, and insect pests (Altaf et al., 2018; Radhakrishnan et al., 2014). Furthermore, *Penicillium* fungi can also exert positive impacts on soil health and ecosystem stability by regulating the structure and function of soil microbial communities (Waqas et al., 2015). It is worth noting that some *Penicillium* strains can dissolve insoluble minerals such as phosphorus and potassium in soil by secreting organic acids including citric acid and oxalic acid, or synthesize siderophores to chelate iron elements in soil, thereby increasing the content of available soil nutrients and improving the nutrient supply status of plants (Zhang et al., 2020). Conversely, the genus *Penicillium* includes well-known plant pathogens that cause postharvest rot and produce mycotoxins. Certain species are also clinically relevant human pathogens. Therefore, the dual nature of this genus necessitates rigorous screening to ensure the non-pathogenicity and biosafety of strains intended for environmental and agricultural applications.

Although the plant growth-promoting potential of *Trichoderma*, *Cladosporium*, and *Penicillium* has been preliminarily confirmed, the molecular mechanisms underlying their positive regulatory effects through interactions with host plants remain unclear, and the differences in growth-promoting mechanisms among strains of different genera have not been systematically elucidated. Based on the above literature, we hypothesized that: (1) endophytic Ascomycota strains from different genera (*Trichoderma*, *Cladosporium*, and Penicillium) can significantly promote the growth of *F. sinensis*, but with distinct effects; (2) these strains promote host growth through divergent molecular pathways, which can be revealed by multi-omics analysis. To fill this research gap and test our hypotheses, this study selected 6 strains of endophytic Ascomycota (classified into the genera *Trichoderma*, *Penicillium*, and *Cladosporium*), which were isolated from high-altitude regions of the Qinghai-Tibet Plateau, as the test strains. Pot experiments were conducted after soaking the seeds of *F. sinensis* with the fungal fermentation broth. First, the growth-promoting effects of each strain were systematically evaluated, and the optimal strains with the best growth-promoting effects within each genus were screened out. Furthermore, metabolomics and transcriptomics technologies were integrated to thoroughly compare the molecular mechanisms by which strains of different genera regulate the growth of *F. sinensis*. This study aimed to: (1) systematically compare the differences in growth-promoting effects of *Trichoderma*, *Cladosporium*, and *Penicillium* strains on *F. sinensis*, and screen high-efficiency growth-promoting strains; (2) compare the core molecular mechanisms of different genera of strains in promoting the growth of *F. sinensis* through multi-omics integrative analysis. (3) Provide theoretical support and strain resources for developing alpine-adapted microbial inoculants to accelerate grassland restoration on the Qinghai-Tibet Plateau.

## 2 Materials and methods

### 2.1 Test strains and forage seeds

Six strains of endophytic Ascomycota (classified into the genera *Penicillium*, *Cladosporium*, and *Trichoderma*), isolated from plants in the high-altitude regions (3,200–4,500 m) of the Qinghai-Tibet Plateau, were selected as the test strains (Supplementary Table 1). The strains used in this study include: *Cladosporium cladosporioides f. pisicola* (Cp3, MZ901118; Cp78, MZ901117), *Penicillium expansum* (Pe305, MZ901031; Pe167, MZ901030), and *Trichoderma alni* (Ta121, MZ901088; Ta227, MZ901093).

Isolation method (Dong et al., 2025a): Fresh roots were collected and surface sterilized with 75% ethanol for 30 s, followed by 5.25% sodium hypochlorite solution for 10 min, and then rinsed five times with sterile water. The roots were then cut into 0.1–0.3 cm fragments and inoculated onto PDA medium (Potato Dextrose Agar, 200 g/L potato, 20 g/L glucose, 17 g/L agar) and incubated in the dark at 20°C for 5–7 days. Marginal mycelia were picked for single colony purification (repeated purification of the strain multiple times), and the species was confirmed through morphological observation and ITS sequence comparison. *F. sinensis* was used as the test forage.

### 2.2 Preparation of strain fermentation broth

Six strains were individually cultured in 500 mL Erlenmeyer flasks containing 200 mL of potato dextrose broth (PDB) liquid medium (inoculation ratio: 10 mL mycelial suspension per 100 mL medium) and incubated on a rotary shaker at 25°C with a shaking speed of 170 r/min for 7 days. Fungal mycelial suspension reached a mycelial dry weight of 0.46 g per 100 mL. During cultivation, growth was monitored, and fermentation broths were collected for subsequent experiments.

### 2.3 Pot experiment design

The seeds of *F. sinensis* were subjected to surface sterilization using a 5% sodium hypochlorite solution. The sterilized seeds were transferred to sterile Petri dishes. Seeds of the forage cultivars were separately soaked in the fermentation broth of a single strain for 6 h at room temperature. Seeds soaked in sterile PDB liquid medium were set as the control group. Upon completion of soaking, 50 plump and intact seeds were selected, evenly placed in sterile petri dishes lined with two layers of sterile filter paper, and moistened thoroughly with 3 mL of sterile water. The seeds were then incubated in a constant-temperature incubator for germination.

The pot experiment was arranged in a completely randomized design with four replications per treatment. A control group (inoculated with sterile water) was included for comparison. The potting medium was prepared by mixing local soil and peat (Klasmann-Deilmann, Germany) at a volume ratio of 5:1. The mixed medium was filled into autoclavable square PP tissue culture bottles, with 600 g of medium per bottle. The medium was sterilized by autoclaving at 121°C for 80 min to eliminate indigenous microorganisms and avoid interference with the inoculated fungal strains. After sterilization, the pots were taken out and cooled to room temperature. Uniform, robust seedlings with a shoot length of approximately 5 cm were selected for transplantation, with 20 seedlings planted per pot. The potted seedlings were cultured under ambient room temperature (20–25°C) with natural light. The growth status of the seedlings was observed and recorded daily, and sterile water was supplemented regularly to maintain moisture.

### 2.4 Collection and processing of forage samples and rhizosphere soil

#### 2.4.1 Collection of forage samples and pretreatment of root samples

At 54 days after seedling transplantation (tillering stage), forage samples and rhizosphere soil were collected from each treatment group. All 20 intact plants from each pot were harvested and evenly divided into two portions: one portion was used for the determination of growth parameters, and the other portion was subjected to the analysis of physiological and biochemical indices, as well as root transcriptome and metabolome profiling.

The roots were first rinsed with tap water to remove the soil adhering to the surface, followed by four rinses with sterile distilled water, and finally blotted dry with sterile filter paper to remove surface moisture. The root samples were aliquoted into cryopreservation tubes, snap-frozen in liquid nitrogen, and then stored in a −80°C refrigerator for subsequent use.

#### 2.4.2 Collection and processing of rhizosphere soil

The collection of rhizosphere soil samples was performed according to the established method (Chen et al., 2021). After harvesting the forage roots, loosely adhering soil was removed by gentle shaking. Subsequently, the soil tightly adhering to the root surface was carefully brushed off using a sterilized brush, and then passed through a 2 mm sieve to eliminate impurities. The collected rhizosphere soil was used for the determination of soil enzyme activities.

### 2.5 Agronomic traits and biochemical determination of forage

Agronomic traits of the forage were measured using vernier calipers and a ruler, including shoot length (from the stem base to the terminal bud) and root length (from the taproot tip to the root collar). Aboveground dry weight was weighed with an electronic balance, and the number of effective tillers per plant (length ≥ 2 cm) was manually counted. Ten plants were measured for each treatment. Leaf physiological traits, including chlorophyll a, chlorophyll b, malondialdehyde (MDA), peroxidase (POD), superoxide dismutase (SOD), proline (PRO), total protein, and catalase (CAT), were quantitatively determined using standardized assay kits (Nanjing Jiancheng Bioengineering Institute, Nanjing, China).

### 2.6 Rhizosphere soil physical and chemical properties and enzyme activity measurement

Rhizosphere soil physical and chemical properties were evaluated via standard chemical analysis methods (Dong et al., 2025a,b): soil organic carbon (SOC, Walkley-Black wet oxidation method), total nitrogen (TN, concentrated H_2_SO_4_ digestion-Kjeldahl method), total phosphorus (TP, HClO_4_-H_2_SO_4_ digestion-molybdenum antimony anti-colorimetric method), available phosphorus (AP, Olsen method with 0.5 mol/L NaHCO_3_ extraction), available nitrogen (AN, alkaline hydrolysis diffusion method), and available potassium (AK, NH_4_OAc extraction-flame photometry).

Enzyme activities in the rhizosphere soil, including amylase (S-AL), urease (S-UE), glucosidase (S-GC), polyphenol oxidase (S-PPO), cellulase (S-CL), and invertase (S-SC), were assayed using commercial enzyme detection kits (Solarbio, Beijing, China). The measurement was performed strictly following the manufacturer’s instructions.

Based on the agronomic traits of the forage, the optimal plant growth-promoting strains were screened out from each genus for further in-depth research, aiming to compare the molecular mechanisms underlying plant growth promotion among different genera.

### 2.7 LC-MS analysis of metabolites in forage roots

A total of 100 mg of root tissues of *Festuca sinensis* cv. Qinghai treated with strains Cp78, Pe167, Ta121 and the CK were weighed for liquid chromatography-mass spectrometry (LC-MS) analysis. For detailed information on the LC-MS analysis, refer to Supplementary material 1.

Metabolomics data were subjected to classification pie chart analysis using the ggplot2 package in R (Wickham, 2011). Orthogonal partial least squares-discriminant analysis (OPLS-DA) was performed with the ropls package, and the model validity was evaluated via cross-validation and permutation tests (Westerhuis et al., 2010). Significantly differential metabolites (SDMs) were screened by combining the variable importance in projection (VIP) values derived from OPLS-DA and the *p*-values obtained from Student’s *t*-test, with the screening thresholds set as VIP > 1, and *p* < 0.05 (Saccenti et al., 2014). Functional annotation and metabolic pathway enrichment analysis of the screened SDMs were conducted via KEGG pathway mapping (Kanehisa and Goto, 2000). K-means clustering analysis was performed using the fuzzy c-means clustering algorithm implemented in the mfuzz package of R. Additionally, a correlation matrix heatmap between clustering modules and agronomic traits was constructed with the psych package in R. The metabolite-metabolite interaction network was built using the online platform MetaboAnalyst 5.0 and visualized with Cytoscape software.

### 2.8 Transcriptomic analysis of forage roots

Root tissues of *Festuca sinensis* cv. Qinghai treated with strains Cp78, Pe167, Ta121 and the CK were selected for total RNA extraction using the mirVana miRNA Isolation Kit (Ambion Inc., Austin, TX, United States). Finally, high-throughput sequencing was performed on the Illumina NovaSeq X Plus platform. For detailed information on the high-throughput sequencing method of root transcriptome, refer to Supplementary material 2.

The assembled transcripts were used as reference sequences. Quality-controlled sequencing reads were aligned to the reference sequences using fastp, and alignment results were summarized with RSeQC (Chen et al., 2018). Transcripts Per Million (TPM) was used to quantify the relative abundance of each transcript in the RNA pool. Significantly differentially expressed genes (DEGs) were identified with the screening criteria set as *q*-value < 0.05 and |Fold Change| > 2 (Robinson et al., 2010). Bar charts depicting the number of DEGs between comparison groups were generated using the ggplot2 package in R (Wickham, 2011). Venn diagram analysis was performed across comparison groups with the VennDiagram package in R to distinguish unique and shared DEGs (Chen and Boutros, 2011). To test whether DEGs were enriched in specific biological functions, gene enrichment analysis was conducted using Fisher’s exact test, and GO enrichment chord diagrams among different comparison groups were plotted with the circlize package in R. KEGG pathway enrichment analysis was performed using clusterProfiler, and bubble charts of enriched KEGG pathways were visualized via the ggplot2 package in R. Metabolic pathways and the interactions among these pathways were illustrated using KEGG pathway maps. Transcription factors (TFs) were annotated by aligning the CDS sequences of DEGs against the transcription factor database. Expression heatmaps of TFs were generated using the pheatmap package in R, and boxplots of the top 20 TF families with the highest expression levels were plotted with the ggplot2 package in R.

### 2.9 Validation by quantitative real-time PCR (RT-qPCR)

To verify the reliability of the RNA sequencing (RNA-seq) data, 9 DEGs were randomly selected for RT-qPCR analysis. Gene-specific primers for the target genes and the reference gene Actin (Accession No. HM623326.1) were designed using Primer Premier 5.0 software, and the primer sequences are provided in Supplementary Table 2. Data normalization was performed using the TPM expression levels of the reference gene (Wu et al., 2019), and the relative expression levels of target genes were calculated using the 2−ΔΔCt method (Okamura et al., 2020).

### 2.10 The ability of strains to produce IAA and inhibit plant pathogens was detected

Indole-3-acetic acid (IAA) production was evaluated using a modified method described by Restu et al. (2023) and quantified by spectrophotometry (Restu et al., 2023). Briefly, 5 mL of fungal spore suspension was inoculated into 100 mL of IAA liquid medium containing peptone (20 g/L), K_2_HPO_4_ (1.5 g/L), MgSO_4_⋅7H_2_O (1.5 g/L), glycerol (10 mL/L), and L-tryptophan (0.1 g/L). The culture was incubated in the dark at 170 rpm for 10 days. At the end of incubation, 2.0 mL of culture was centrifuged at 13,000 rpm for 5 min and filtered through a 0.22 μm filter. Then, 1 mL of supernatant was mixed with 2 mL of Salkowski reagent (0.5 mol/L FeCl_3_ and 35% perchloric acid), kept in the dark at room temperature for 30 min, and the absorbance was measured at 530 nm. The IAA concentration was calculated based on a standard curve. Samples were collected at 3, 4, 5, 6, 7, 8, 9, and 10 days with seven replicates. The antagonistic ability of each strain against plant pathogens was determined using the plate confrontation method.

### 2.11 Statistical analysis

All data were expressed as mean ± standard deviation (SD). According to the characteristics of data distribution, statistical tests were performed using one-way analysis of variance (one-way ANOVA) with the significance level set at *p* < 0.05. Data analysis was completed using SPSS Statistics 26.0 software. The interaction network among SDMs, DEGs, and TFs was constructed and visualized using Cytoscape 3.8.2 software.

## 3 Results

### 3.1 Responses of *F. sinensis* and its rhizosphere soils metabolism to fungal strains of different genera

The physicochemical properties of the mixed potting medium before sowing were as follows: total nitrogen (TN) 1.88 ± 0.05 g/kg, alkali-hydrolyzable nitrogen (AN) 221.33 ± 4.04 mg/kg, total phosphorus (TP) 2.04 ± 0.03 g/kg, available phosphorus (AP) 75.97 ± 0.35 mg/kg, soil organic carbon (SOC) 29.57 ± 0.38 g/kg, and available potassium (AK) 253.67 ± 2.31 mg/kg (Supplementary Table 3).

Phylogenetic analysis of the six endophytic Ascomycete strains was performed based on ITS sequences using MEGA 11 software. The resulting phylogenetic tree (Supplementary Figure 1) showed that all strains were clearly divided into three distinct clades with 100% bootstrap support. Specifically, the two *Trichoderma* isolates (Ta227, MZ901093; Ta121, MZ901088) and two *Cladosporium* isolates (Cp78, MZ901117; Cp3, MZ901118) were closely related and clustered into a single major clade, whereas the two *Penicillium* isolates (Pe305, MZ901031; Pe167, MZ901030) formed an independent, well-supported clade.

**FIGURE 1:**
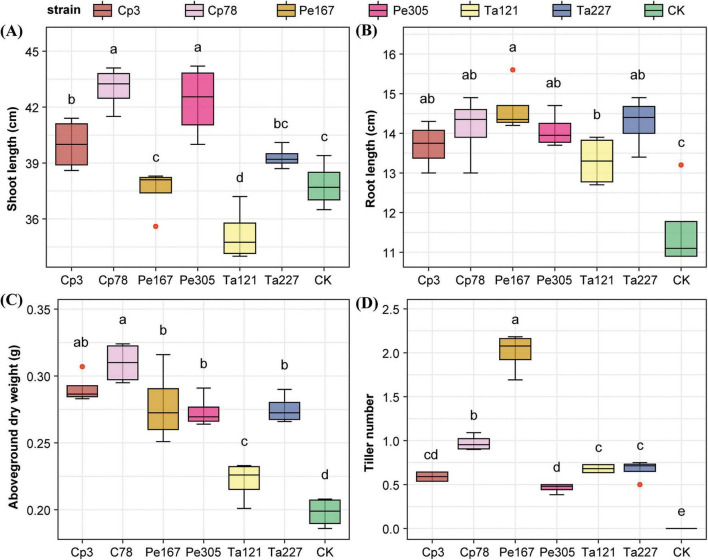
Regulatory effects of strain treatments on the growth of *Festuca sinensis* cv. Qinghai. **(A)** Shoot length; **(B)** root length; **(C)** aboveground biomass; **(D)** tiller number. Statistical analysis was performed using Student’s *t*-test. Different lowercase letters above the boxplots indicate significant differences (*p* < 0.05), *n* = 4.

Compared with CK, *F. sinensis* treated with different strains exhibited superior growth performance, with intraspecific variations in the growth-promoting effects among strains. Meanwhile, fungal strain treatments significantly regulated the contents of physiological active substances in plants and enzyme activities in rhizosphere soils. Specifically, Cp78 and Cp3 as well as Pe305 significantly increased the shoot length of *F. sinensis*, with respective increments of 13.75, 5.75, and 11.90% (Figure 1A). In addition, all tested strains significantly increased the root length and aboveground dry weight of the forage, and the growth-promoting effects varied among different strains within the same genus: Cp78 outperformed Cp3; Pe167 was more effective than Pe305; no significant difference was observed between Ta121 and 227 (Figures 1B,C). Notably, strain treatments effectively accelerated the growth period progression of *F. sinensis*. When the CK group had not yet entered the tillering stage, all strain-treated groups had successfully tillered (Figure 1D). The promoting effect on tillering was consistent with that on root length and aboveground biomass: Cp78 was superior to Cp3, Pe167 was better than Pe305, and there was no significant difference between Ta121 and Ta227.

Furthermore, strain treatments significantly altered the antioxidant enzyme activities (Supplementary Figures 2A–D), osmotic adjustment substance contents (Supplementary Figures 2E,F) and chlorophyll contents (Supplementary Figures 2G,H) in the leaves of *F. sinensis*, with distinct differences in regulatory effects among different strains. A similar pattern was observed in the rhizosphere soil enzyme activities: strain treatments significantly affected soil enzyme activities, with notable inter-strain differences (Supplementary Figure 3). Based on the comprehensive evaluation of multiple phenotypic and physiological indicators, including growth traits, antioxidant capacity, photosynthetic pigment contents, and rhizosphere soil enzyme activities, the most effective strain from each genus was selected for further in-depth omics analysis: Cp78, Pe167, and Ta121. These three strains exhibited the strongest overall growth-promoting effects within their respective genera. Accordingly, forage samples from these three treatments were used for subsequent transcriptomic and metabolomic analyses to elucidate the molecular mechanisms underlying the growth promotion of *F. sinensis* by different endophytic fungal genera.

### 3.2 Identification of root metabolites in *F. sinensis* following fungal strain seed priming

To explore the responses of root metabolites to different treatments, untargeted metabolomic profiling was performed on the roots of *F. sinensis* treated with strains Ta121, Cp78, Pe167, and a control (CK). Principal component analysis (PCA) results (Supplementary Figures 4A,B) showed that the four treatment groups were clearly separated under both positive and negative ion modes, with tight clustering of biological replicates within each group. These findings indicated that different strain treatments significantly altered the root metabolic profiles of *F. sinensis*, and distinct metabolic characteristics were observed between the treated groups and the control. After quality control, a total of 1,430 metabolites were identified, including 824 in positive ion mode and 606 in negative ion mode (Supplementary Table 4). These metabolites were categorized into 13 classes, with the top five most abundant classes being lipids and lipid-like molecules (30.7%), phenylpropanoids and polyketides (15.9%), organic acids and derivatives (15.3%), organoheterocyclic compounds (11.8%), and benzenoids (9.1%) (Supplementary Figure 5).

**FIGURE 2:**
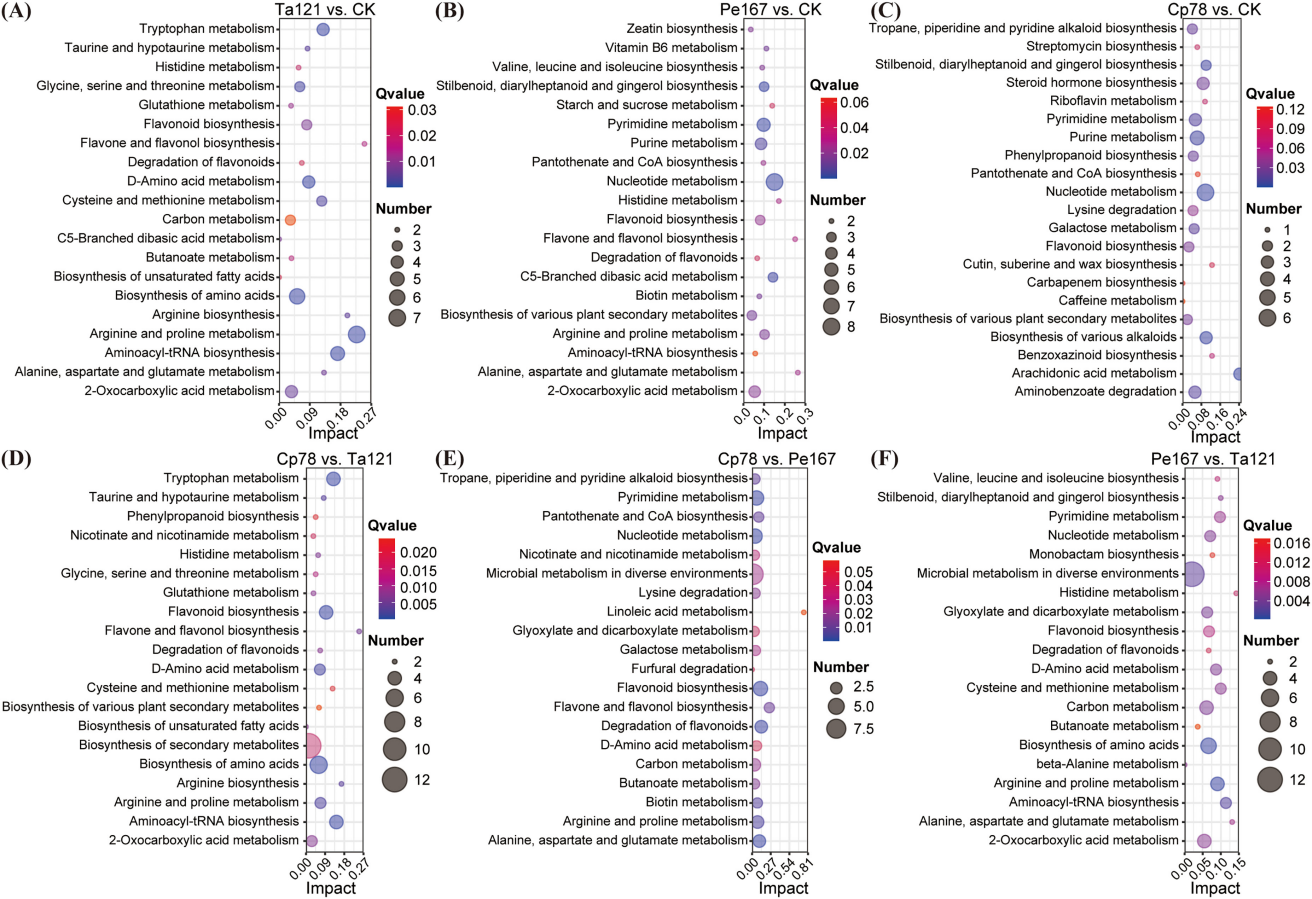
KEGG pathway enrichment analysis of significantly differential metabolites (SDMs) in different comparison groups. **(A)** Ta121 vs. CK group; **(B)** Pe167 vs. CK group; **(C)** Cp78 vs. CK group; **(D)** Cp78 vs. Ta121 group; **(E)** Cp78 vs. Pe167 group; **(F)** Pe167 vs. Ta121 group.

A total of 713 SDMs were identified (Supplementary Tables 5–7). The KEGG pathway enrichment analysis of these SDMs revealed variations in the number of significantly enriched pathways (Impact > 0.1 and Qvalue < 0.05) among different comparison groups: 7 pathways in Ta121 vs. CK, 10 in Pe167 vs. CK, 3 in Cp78 vs. CK, 6 each in Ta121 vs. Cp78 and Ta121 vs. Pe167, and 5 in Pe167 vs. Cp78 (Figures 2A–F). The enrichment profiles exhibited distinct genus-specific characteristics: comparison groups involving strain Ta121 shared 5 enriched pathways including tryptophan metabolism; groups involving strain Pe167 were Flavonoid biosynthesis, with zeatin biosynthesis pathway specifically enriched in Pe167 vs. CK; the Cp78 vs. CK group was enriched biosynthesis of various alkaloids.

**FIGURE 3:**
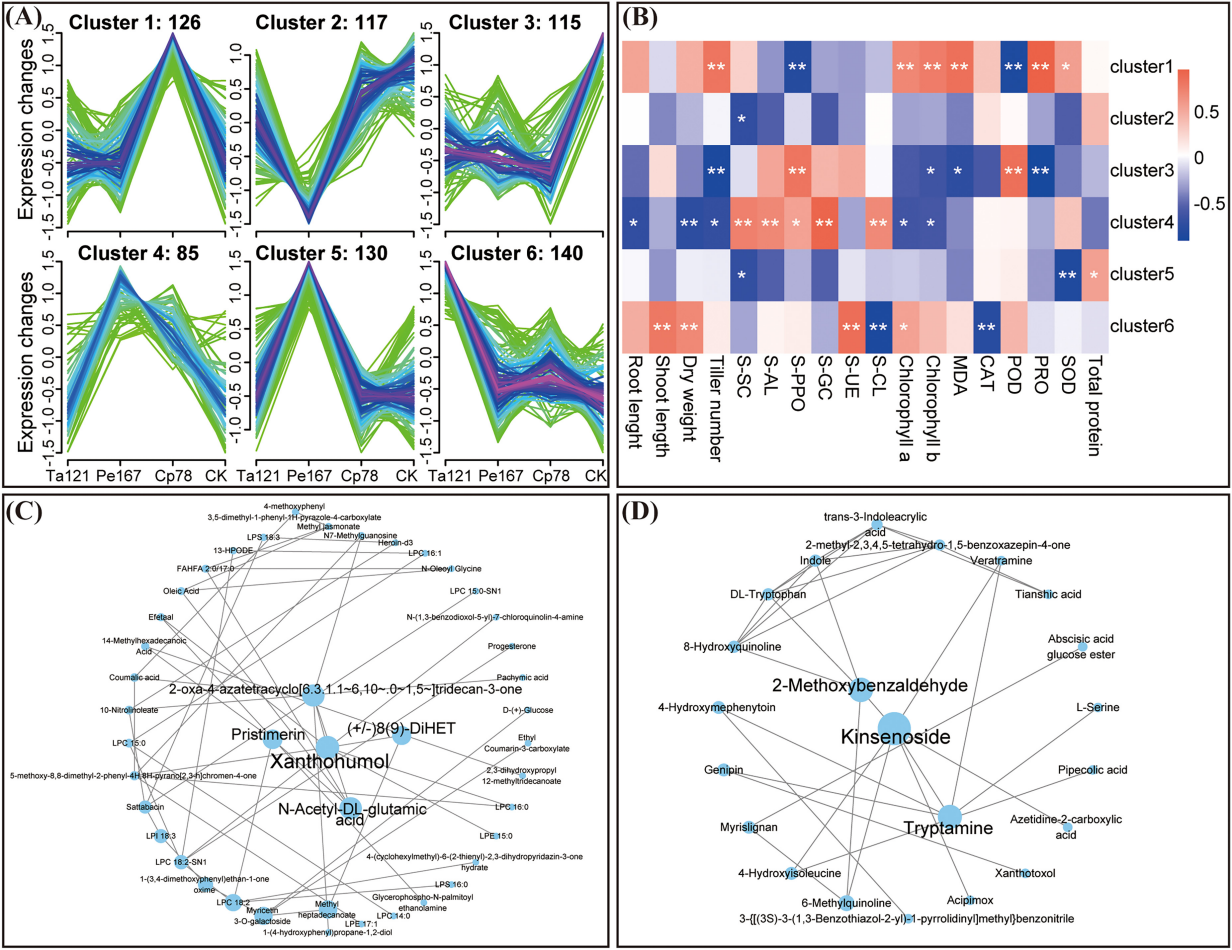
K-means clustering, correlation analysis, and interaction networks of significantly differential metabolites (SDMs). **(A)** K-means clustering map of 713 significantly differential metabolites; **(B)** correlation heatmap between the variation trends of metabolite clustering modules and forage growth and physiological indices; **(C,D)** metabolite-metabolite interaction networks of SDMs in clusters 1–3 **(C)** and clusters 1–6 **(D)**. Stars indicate the significance level of Pearson correlation coefficients (**p < 0.01; *0.01 < *p* < 0.05).

Results of K-means clustering analysis showed that the 6 distinct clusters of SDMs corresponded to the 4 different treatment groups: Cp78 (cluster 1), Pe167 (clusters 4 and 5), Ta121 (cluster 6), and CK (clusters 2 and 3) (Figure 3A). This indicated that the accumulation patterns of the SDMs in the roots of forage treated with strains of different genera were diverse. Correlation analysis results (Figure 3B) revealed that the SDMs in cluster 1 were significantly positively correlated with forage tillering, chlorophyll content, and antioxidant indicators (SOD, PRO, and MDA). The SDMs in cluster 4 were significantly positively correlated with soil enzymes (S-CL, S-GC, S-SC, S-AL, and S-PPO). The SDMs in cluster 5 were correlated with total protein content. The SDMs in cluster 6 were significantly positively correlated with growth parameters (chlorophyll a content, shoot length, and dry weight) and S-UE activity. The SDMs in cluster 3 were only significantly positively correlated with antioxidant enzymes (POD and S-PPO). In summary, *Cladosporium* primarily regulated rhizosphere metabolites to exert positive effects on forage tillering, chlorophyll synthesis, and antioxidant enzyme activation. *Penicillium* modulated root metabolites to positively affect soil enzyme activities in the forage rhizosphere. In contrast, *Trichoderma* promoted forage growth, chlorophyll accumulation, and plant utilization of rhizospheric nitrogen nutrients by regulating root metabolites.

To further clarify the effects of strain priming on the root metabolic profiles of forages, metabolite-metabolite interaction networks were constructed separately using cluster 1 and 6 (associated with growth) as well as cluster 1 and 3 (associated with antioxidant enzyme activities) (Figures 3C,D). The results showed that the core SDMs in cluster 1–3 were xanthohumol and pristimerin, which were mainly involved in the flavonoid biosynthesis pathway; the core SDMs in cluster 1–6 were kinsenoside, tryptamine, and 2-methoxybenzaldehyde. The metabolic pathways involving these core SDMs were completely consistent with the significantly enriched pathways identified in each comparison group.

### 3.3 Identification of DEGs in *F. sinensis* roots under strain treatments

RNA sequencing (RNA-seq) was performed to analyze the gene expression profiles of *F. sinensis* treated with strains Ta121, Cp78, Pe167, and CK. High-quality clean data were obtained for all treatment groups (Supplementary Table 8). The raw read counts of the four groups were 55,236,178, 56,975,888, 47,894,810, and 53,010,568, respectively. The Q20 values were no < 98.18%, Q30 values were ≥ 94.67%, and the GC content ranged from 57.45 to 58.61%, indicating the high reliability of the sequencing data. After filtering low-expression transcripts, a total of 563,233 transcripts and 217,233 unigenes were obtained with good assembly quality (Supplementary Table 9). All sequencing data have been deposited in the NCBI database (accession no.: PRJNA1164999).

**FIGURE 4:**
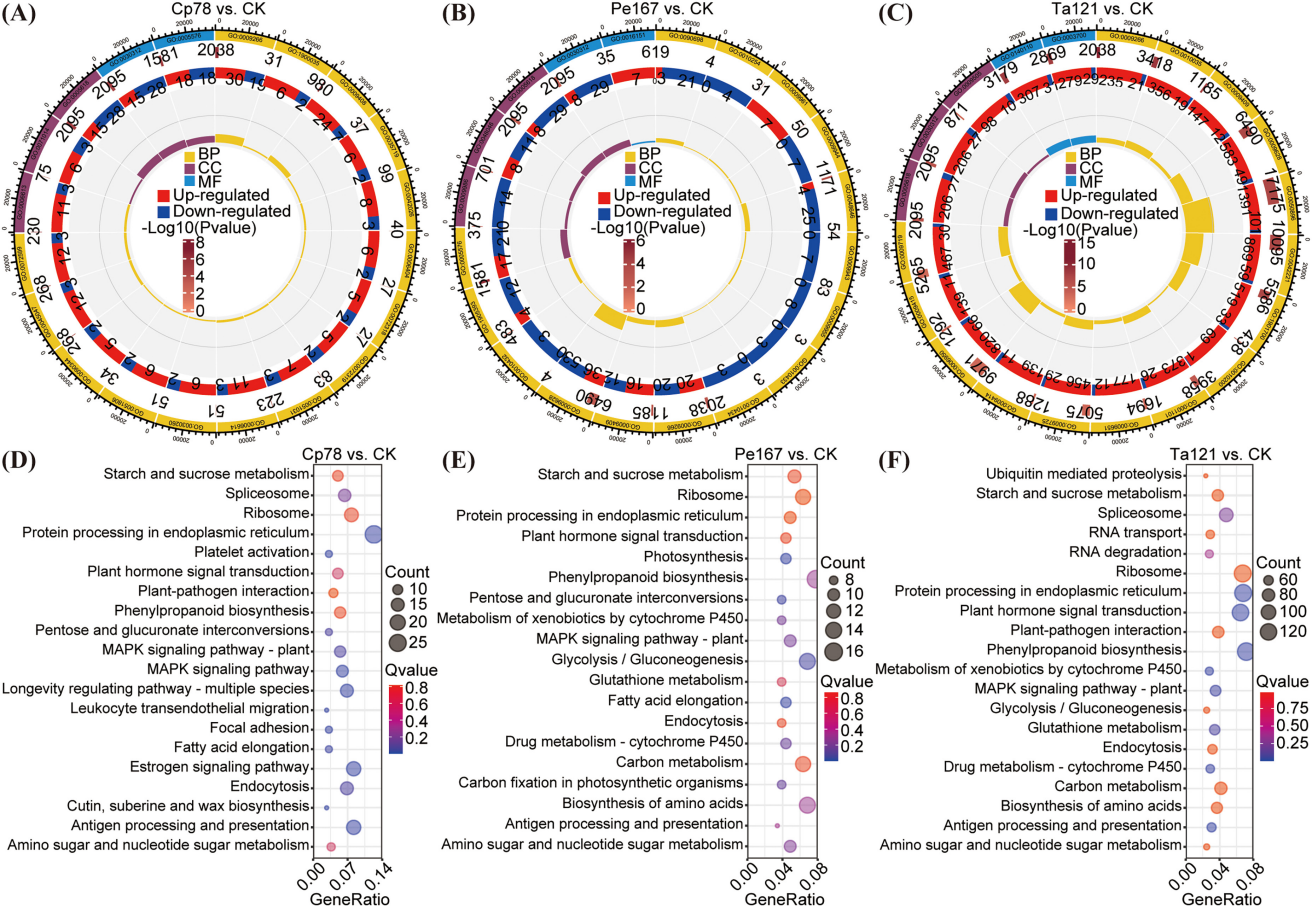
Enrichment analysis of differentially expressed genes (DEGs) in different comparison groups. **(A–C)** Gene Ontology (GO) functional enrichment of DEGs in the Cp78 vs. CK **(A)**, Pe167 vs. CK **(B)**, and Ta121 vs. CK **(C)** groups; **(D**–**F)** KEGG pathway enrichment analysis of DEGs in the Cp78 vs. CK **(D)**, Pe167 vs. CK **(E)**, and Ta121 vs. CK **(F)** groups.

A total of 217,223 genes expressed in at least one sample were detected. A total of 13,234 DEGs were identified, specifically, 11,871 DEGs (11,070 up-regulated and 801 down-regulated) in the Ta121 vs. CK group, 1,656 DEGs (714 up-regulated and 942 down-regulated) in the Cp78 vs. CK group, and 1,569 DEGs (638 up-regulated and 931 down-regulated) in the Pe167 vs. CK group (Supplementary Figure 6A). Venn diagram analysis revealed 444 common DEGs among the three comparison groups (Supplementary Figure 6B). Gene Ontology (GO) functional analysis indicated that these DEGs were enriched in the categories of molecular function, cellular component and biological process (Figures 4A–C). The KEGG pathway enrichment analysis demonstrated that the enriched pathways were strain-specific (Figures 4D–F). Specifically, DEGs in the Ta121 vs. CK group were significantly enriched in 7 pathways including plant hormone signal transduction, phenylpropanoid biosynthesis and MAPK signaling pathway; DEGs in the Cp78 vs. CK group were enriched in 13 pathways such as estrogen signaling pathway, MAPK signaling pathway, and cutin, suberine and wax biosynthesis; DEGs in the Pe167 vs. CK group were enriched in 4 pathways including photosynthesis, and glycolysis/gluconeogenesis.

Nine DEGs were selected for expression validation using quantitative real-time polymerase chain reaction (qRT-PCR). The results showed that their expression trends were consistent with those of the RNA-seq data (Supplementary Figure 7), confirming the reliability of the sequencing results.

### 3.4 Analysis of DEGs in distinct biosynthetic pathways

**FIGURE 5:**
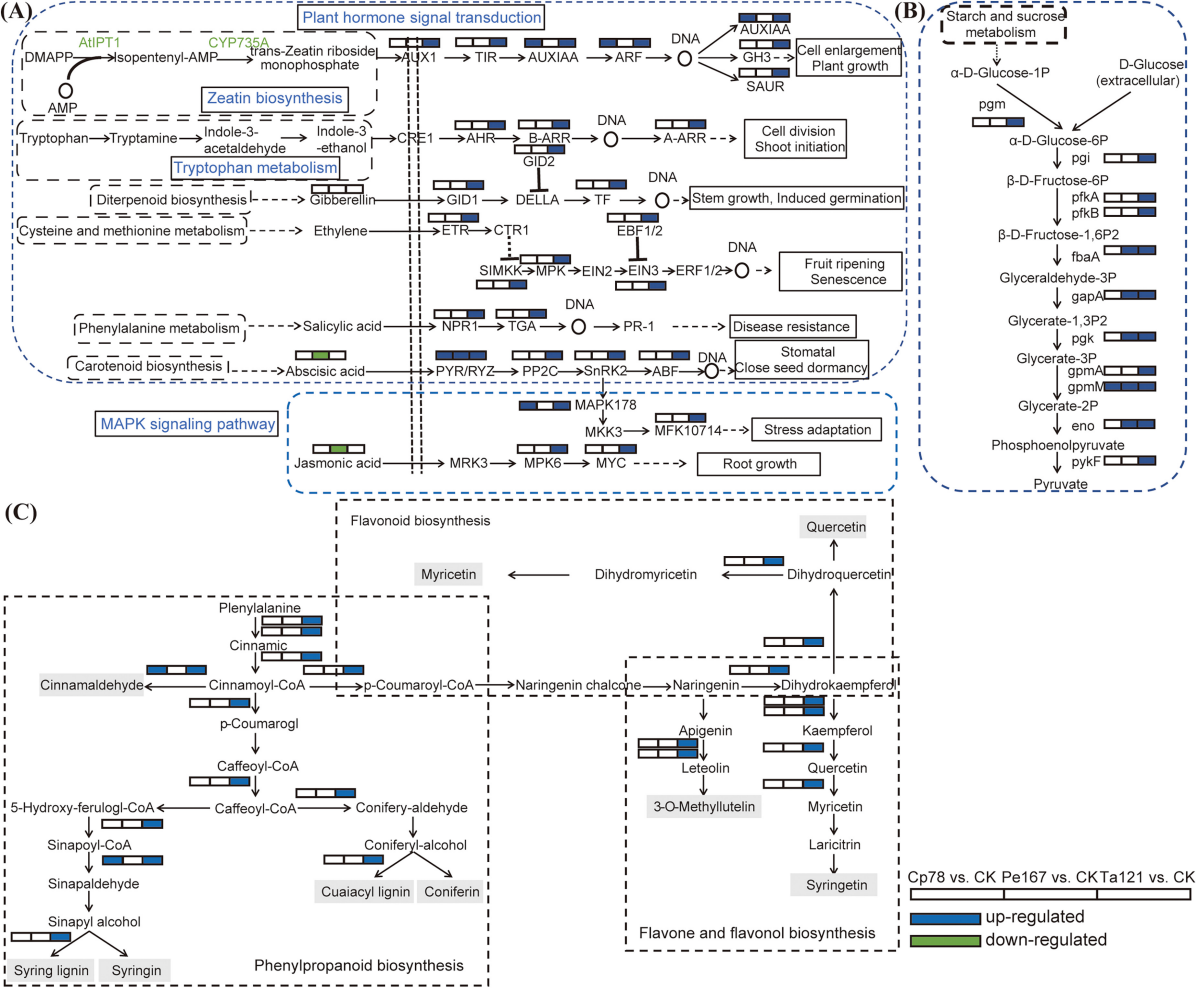
Expression trends of differentially expressed genes (DEGs) and significantly differential metabolites (SDMs) in six biosynthetic pathways across different comparison groups. **(A)** Plant hormone signal transduction pathway and MAPK signaling pathway; **(B)** glycolysis/gluconeogenesis pathway; **(C)** Phenylpropanoid biosynthesis pathway, flavonoid biosynthesis pathway, and flavonoid and flavonol biosynthesis pathway. Different grids represent distinct comparison groups, ordered as Cp78 vs. CK, Pe167 vs. CK, and Ta121 vs. CK, sequentially. In terms of grid fill colors, red indicates up-regulation, while blue indicates down-regulation.

To clarify the molecular mechanisms underlying the up- and down-regulation of SDMs in different treatment groups, DEGs within the significantly enriched pathways were analyzed. In the plant hormone signal transduction pathway, a total of 24 DEGs were identified (Figure 5A), and the gene expression patterns varied distinctly among different strain-treated groups. Specifically, all 24 DEGs, covering 22 gene families including AUX1, TIR, and AUXIAA, were significantly up-regulated in the Ta121 vs. CK group; 4 DEGs (2 AUXIAA, 1 ARF, and 1 PYR) were significantly up-regulated in the Cp78 vs. CK group; while only the PYR gene was significantly up-regulated in the Pe167 vs. CK group. A total of 11 DEGs were detected in the glycolysis/gluconeogenesis pathway (Figure 5B). All 11 core genes (including pgm, pgi and pfkA) were significantly up-regulated in the Ta121 vs. CK group; 5 DEGs were significantly up-regulated in the Pe167 vs. CK group; whereas only the gpmM gene was significantly up-regulated in the Cp78 vs. CK group. Analysis of DEGs involved in phenylpropanoid, flavonoid, flavone and flavonol biosynthesis pathways showed that the Ta121 vs. CK group had the largest number of up-regulated genes, including 12 genes in the phenylpropanoid biosynthesis pathway, 7 in the flavonoid and flavonol biosynthesis pathway, and 3 in the flavonoid biosynthesis pathway (Figure 5C). By contrast, only 2 genes related to the phenylpropanoid biosynthesis pathway were up-regulated in the Cp78 vs. CK group.

### 3.5 Analysis of DEGs encoding transcription factors

Transcription factors are key regulators of plant growth and development. In this study, a total of 335 TFs were identified from the DEGs, which were classified into 41 families. The total TPM values of TFs in different families varied among the treatment groups (Supplementary Figure 8A). Among them, 10 dominant TF families contained no < 10 members, including ERF (49), WRKY (27), and bHLH (26 members). Compared with CK, the total TPM values of most TF families in the Ta121 strain-treated group were higher, with the exception of 5 families including B3 and GRF. Based on the average TPM values across the four treatment groups, the top 20 TF families in terms of expression level were screened (Supplementary Figure 8B). The results showed that the expression level of the ERF family was 2.65-fold higher than that of other families, exhibiting the highest average expression level. This was followed by the WRKY and C2H2 families, while the GeBP family showed the lowest average expression level. In addition, the NF-YA, AP2, and YABBY families displayed comparable average expression levels.

### 3.6 Multi-omics integrated analysis of key metabolites, core genes, and TFs regulating *F. sinensis* growth promoted by three fungal genera

**FIGURE 6:**
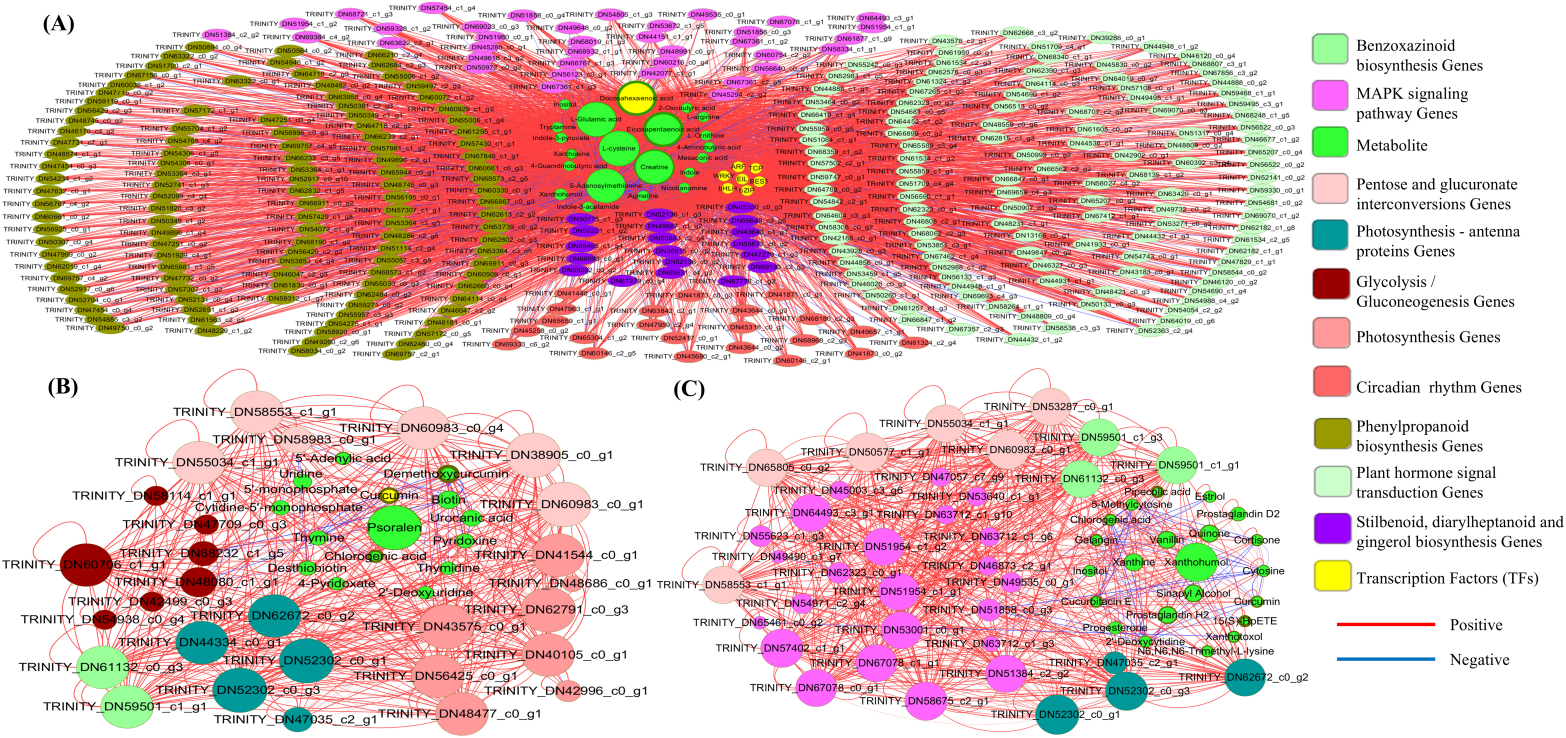
Correlation network analysis of key metabolites, core genes, and transcription factors in pathways enriched by three fungal genera. **(A)** Association network of differentially expressed genes (DEGs), significantly differential metabolites (SDMs), and transcription factors (TFs) in the significantly enriched pathways of the *Trichoderma* group; **(B)** that of the *Penicillium* group; **(C)** that of the *Cladosporium* group. Different colored nodes represent different types of molecules; node size reflects the number of SDMs or DEGs significantly correlated with the node; the color of edges between nodes defines the direction of correlation: red indicates significant positive correlation, and blue indicates significant negative correlation; edge thickness corresponds to the absolute value of the correlation coefficient.

To identify the key metabolites, core genes, and TFs mediating the growth-promoting effects of *Trichoderma* (Ta121), *Cladosporium* (Cp78), and *Penicillium* (Pe167) in *F. sinensis*, interaction networks were constructed using DEGs, SDMs, and TFs derived from the significantly enriched pathways (Figure 6). The results demonstrated that the molecular mechanisms of growth regulation varied significantly among the fungal genera, with the regulatory network of *Trichoderma* being notably more complex. Detailed genus-specific analyses are presented as follows:

For the *Trichoderma* group (Figure 6A), association network analysis was performed on DEGs, TFs, and corresponding SDMs involved in five pathways including plant hormone signal transduction and phenylpropanoid biosynthesis. Six SDMs (docosahexaenoic acid, eicosapentaenoic acid, creatine, L-cysteine, L-glutamic acid, and S-adenosylmethionine) showed significant correlations with multiple DEGs, among which only S-adenosylmethionine exhibited a negative correlation. Docosahexaenoic acid, eicosapentaenoic acid, and creatine had higher fold changes (FC) compared with other SDMs. Six TF families (ARF, BES1, bHLH, bZIP, EIL, and WRKY) were positively correlated with five SDMs, whereas TCP was negatively correlated with four SDMs. The plant hormone signal transduction and phenylpropanoid biosynthesis pathways contained the largest number of DEGs (123 and 136, respectively). These findings suggest that *Trichoderma* may regulate plant growth through the synergistic modulation of plant hormone signal transduction and phenylpropanoid biosynthesis pathways, coupled with interactions between multiple TF families and SDMs. The involvement of more pathways and molecules than other genera further confirms the complexity of its regulatory mechanism.

For the *Penicillium* group (Figure 6B), association networks were established using molecules from five pathways including glycolysis/gluconeogenesis and photosynthesis. Psoralen was positively correlated with multiple DEGs, while demethoxycurcumin, curcumin, and chlorogenic acid had higher FC values. The glycolysis/gluconeogenesis pathway contained the largest number of DEGs (11), among which the gene TRINITY_DN60706_c1_g1 was positively correlated with multiple DEGs and SDMs. These data suggest that the glycolysis/gluconeogenesis pathway serves as the regulatory core of *Penicillium*, which promotes plant growth by regulating carbon metabolism through interactions between genes in this pathway and specific SDMs.

For the *Cladosporium* group (Figure 6C), interaction networks were constructed using molecules from four pathways including the MAPK signaling pathway and photosynthesis-antenna proteins. Xanthohumol was significantly positively correlated with multiple DEGs, while 15(S)-hydroxyeicosatetraenoic acid and pipecolic acid showed higher FC values. The MAPK signaling pathway contained the maximum number of DEGs (23), among which 8 genes were positively correlated with multiple DEGs and SDMs. These results indicate that *Cladosporium* primarily exerts its growth-regulating effects via the MAPK signaling pathway, relying on the synergistic interactions between core SDMs and genes in this pathway.

## 4 Discussion

This study is the first to integrate the phylogenetic relationships of endophytic ascomycetes with their growth-promoting effects on *F. sinensis*, revealing a significant correlation between these two aspects. Phylogenetic analysis indicated that *Trichoderma* and *Cladosporium* strains are closely related and clustered into the same evolutionary clade, whereas *Penicillium* formed an independent clade. This phylogenetic characteristic was completely consistent with the complexity gradient of the strains’ growth-promoting mechanisms (*Trichoderma* sp. Ta121 > *Cladosporium* sp. Cp78 > *Penicillium* sp. Pe167) (Supplementary Figure 1), suggesting that the evolutionary status of the strains may be closely associated with the differentiation of their growth-promoting functions. These findings provide a novel theoretical basis for the targeted screening of high-efficiency growth-promoting strains suitable for alpine regions. During long-term evolution, *Trichoderma*, *Cladosporium*, and *Penicillium* have developed distinct growth-promoting mechanisms.

### 4.1 *Trichoderma* sp. Ta121: a dual growth-promoting mechanism mediated by synergistic regulation of multiple hormone signaling pathways

The symbiotic relationship between *Trichoderma* and plants has been widely verified, and one of its core growth-promoting mechanisms is to directly regulate plant growth and enhance nutrient uptake efficiency through the synthesis of plant hormones (Contreras-Cornejo et al., 2016; Contreras-Cornejo et al., 2024; Woo et al., 2023). This study confirmed that Ta121 has the ability to synthesize indole-3-acetic acid (IAA), with the IAA concentration in the fermentation broth reaching 6.03 μg/mL on the 10th day of cultivation, which is comparable to that of the well-known plant growth-promoting *Trichoderma* sp. DK (Supplementary Figure 9). Further metabolomic analysis revealed that *Trichoderma* treatment significantly enriched the tryptophan metabolism pathway, which represents the core biosynthetic pathway for endogenous plant auxins and their precursors (Figure 2A). Transcriptomic data indicated that the key genes involved in the auxin signaling pathway (AUX1, TIR1, AUX/IAA, ARF, GH3, SAUR) were significantly up-regulated in the treatment group (Figure 5A). Combined with phenotypic data, the *Trichoderma* strain used in this study significantly promoted biomass accumulation, root elongation, and tiller germination in *F. sinensis*, confirming that IAA synthesis and signal regulation are the core growth-promoting pathways of this strain. This result is consistent with previous studies demonstrating that *Trichoderma*-derived IAA can significantly promote the growth of plants such as *Arabidopsis thaliana*, *Syringa oblata*, and *Capsicum annuum* (Bader et al., 2020; Illescas et al., 2021; Jin et al., 2023; Martínez-Medina et al., 2014; Nieto-Jacobo et al., 2017; Saber et al., 2017).

**FIGURE 7:**
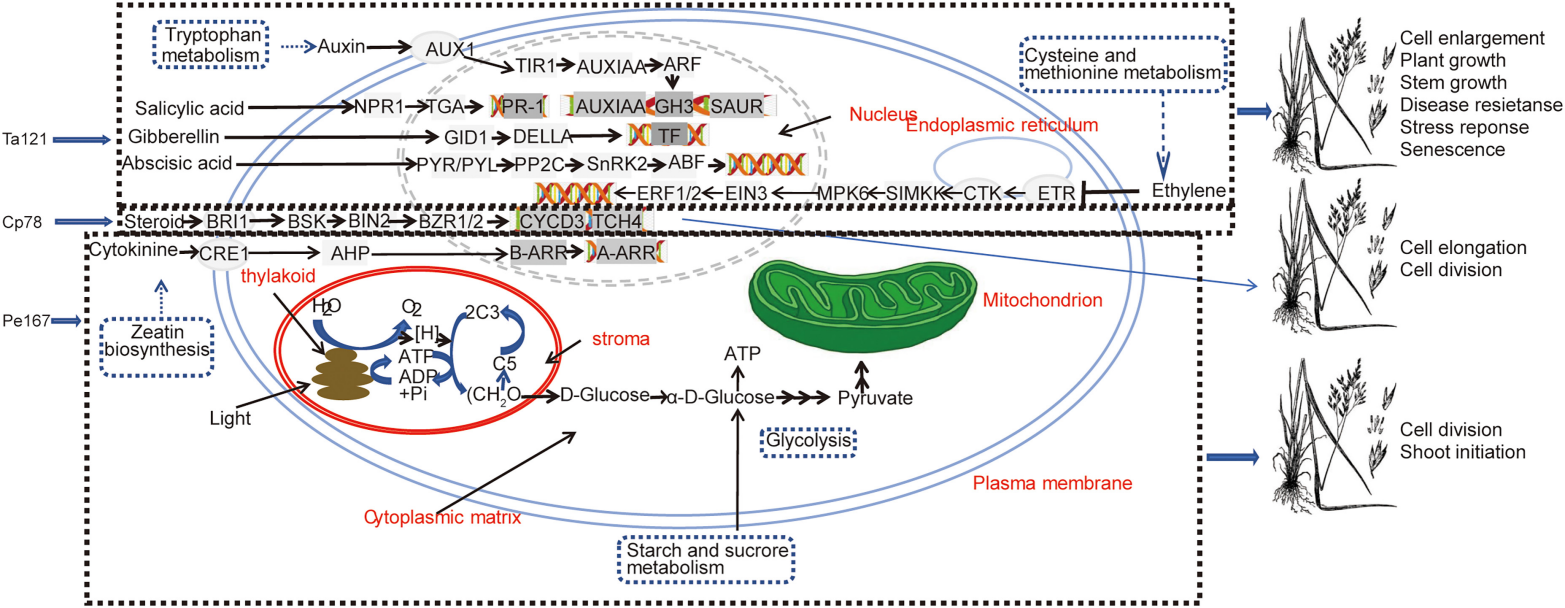
Schematic diagram of the molecular mechanisms underlying growth promotion in *Festuca sinensis* cv. Qinghai by seed soaking with strains of three fungal genera.

In addition, *Trichoderma* exerts dual effects of plant growth promotion and stress tolerance by regulating other hormone signaling pathways. First, it significantly enriched the cysteine and methionine metabolism pathway (a precursor pathway for ethylene synthesis) and up-regulated the expression of the ethylene receptor gene ETR and the signaling pathway gene MPK6 (Figure 7). Ethylene signaling is known to promote plant root hair development and lateral root formation (Iqbal et al., 2017). Second, it up-regulated the expression of NPR1 and TGA, the key genes in the salicylic acid signaling pathway (Figure 5A). This is consistent with the conclusion that *Trichoderma* can induce systemic resistance in plants by activating the salicylic acid signaling pathway, thereby enhancing disease resistance (Kredics et al., 2024). Third, it significantly increased the content of abscisic acid glucose ester (ABA-GE, an inactive precursor of abscisic acid), which can be converted into active abscisic acid via β-glucosidase to improve plant stress tolerance (Lee et al., 2006; Liu et al., 2015; Xu et al., 2012). In summary, *Trichoderma* triggers signal cascade responses in host plants through the synergistic regulation of multiple hormone signaling pathways including auxin, ethylene, salicylic acid, and abscisic acid, ultimately achieving dual effects of growth promotion and stress tolerance enhancement. This is consistent with the general characteristic of *Trichoderma* exerting growth-promoting effects through plant hormone synthesis (Alizadeh et al., 2024; Galeano et al., 2024).

### 4.2 *Cladosporium* sp. Cp78: a distinctive mechanism involving brassinosteroid pathway regulation and antimicrobial metabolite synthesis

Whole-genome studies have demonstrated that the *Cladosporium* genome contains gene clusters related to sterol biosynthesis, and sterols are the precursor substances of brassinosteroids (BRs), a class of key growth-regulating hormones (Li et al., 2021; Lian et al., 2023). This study found that *Cladosporium* treatment significantly enriched the steroid hormone biosynthesis pathway, and transcriptomic data showed that the expression of BRI1, the key gene encoding the brassinosteroid signal receptor, was significantly up-regulated (Figure 7). This gene mediates the transduction of extracellular BR signals into cells, promoting plant cell elongation and division, thus confirming that BR pathway regulation is the core growth-promoting mechanism of *Cladosporium* (Li et al., 2021). In addition, *Cladosporium* treatment significantly enriched the biosynthetic pathways of alkaloids, phenylpropanoids, and flavonoids (Figure 2C). Alkaloids and their derivatives can inhibit pathogen growth, while flavonoids enhance plant stress tolerance (Wu et al., 2023). Pathogen antagonism assays also confirmed that *Cladosporium* exhibited significant inhibitory effects against *Alternaria* spp. (DT-DYLC, DT-XRKA) and *Fusarium fujikuroi* (DT-08C) (Supplementary Figure 9B), suggesting that antimicrobial metabolite synthesis is another important mechanism for *Cladosporium* to promote plant growth, which indirectly ensures host growth vigor by reducing disease stress.

### 4.3 *Penicillium* sp. Pe167: growth-promoting mechanism mediated by carbon metabolism optimization

The unique growth-promoting advantage of *Penicillium* lies in its ability to interact with plant cells by releasing beneficial compounds and regulate plant metabolic processes (Khan et al., 2008; Lu et al., 2019). In this study, *Penicillium* treatment significantly enriched the starch and sucrose metabolism, photosynthesis, and glycolysis pathways, suggesting that this strain may improve plant carbon cycling processes to provide sufficient material and energy for robust plant growth and development (Figure 4E). Pathogen antagonism assays confirmed that *Penicillium* exhibited antagonistic activity against three common plant pathogens (Supplementary Figure 9B), which is consistent with the disease-control mechanism of *Penicillium* inducing pathogen hyphal deformation by secreting alkaloids and terpenoids (Ai et al., 2023; Attia et al., 2022). Metabolomic analysis further verified this conclusion: after *Penicillium* treatment, the contents of phenylpropanoids, polyketides, alkaloids, and their derivatives in the roots of forage increased significantly (Supplementary Figure 5C), and these substances have been proven to possess antimicrobial activity (Li et al., 2021).

Meanwhile, *Penicillium* exerts growth-promoting effects through hormone regulation and nutrient solubilization. First, it significantly enriched the zeatin biosynthesis pathway and regulated plant growth and development by stimulating the secretion of endogenous auxins (Figure 2B). This is consistent with studies showing that *Penicillium* synthesizes auxins to regulate plant root development and improve nutrient conversion efficiency (Lafuente and Gonzalez-Candelas, 2024). Phenotypic data also showed that the root length and tiller number of forage in the *Penicillium* treatment group reached the maximum, further confirming its plant growth-promoting regulatory effect. Second, it solubilizes insoluble soil phosphorus by secreting acidic substances. Metabolomic analysis indicated that *Penicillium* treatment significantly increased the contents of organic oxygen-containing compounds, organic acids, and their derivatives in the roots of forage (Supplementary Figure 5C). These substances can decompose inorganic and organic insoluble phosphorus in soil, thereby increasing the content of available soil phosphorus and improving soil fertility (Suraby et al., 2023). This mechanism provides critical nutrient support for forage growing in the nutrient-poor soils of the Qinghai-Tibet Plateau.

### 4.4 Commonalities and specificities of growth-promoting mechanisms among strains of different genera

Based on the above analysis, the growth-promoting mechanisms of *Trichoderma*, *Cladosporium* and *Penicillium* exhibit significant commonalities and specificities. The commonalities are reflected in the realization of growth-promoting effects through hormone regulation and antimicrobial metabolite synthesis, as well as the adaptation to the growth requirements of forage in the alpine environment of the Qinghai-Tibet Plateau. Notably, despite their distinct core mechanisms, all three strains share key adaptive features that enhance the resilience of *F. sinensis* to the harsh conditions of the Qinghai-Tibet Plateau. This aligns with the growing body of literature demonstrating that beneficial endophytes from extreme environments often evolve convergent strategies to promote host fitness under abiotic stress (Dong et al., 2025a,b; Morales-Vargas et al., 2024). For example, the synthesis of antimicrobial metabolites (alkaloids, flavonoids, and phenylpropanoids) by all three strains not only protects the forage from pathogenic infections but also helps shape a beneficial rhizosphere microbiome, a critical adaptation in nutrient-poor and pathogen-prone alpine soils (Ramaroson et al., 2022; Wankhade et al., 2025). Similarly, the regulation of hormone signaling pathways (auxin, cytokinin, and brassinosteroids) by these fungi directly counteracts the growth-limiting effects of low temperatures and nutrient scarcity, a common strategy employed by psychrotolerant endophytes to promote plant growth in cold ecosystems (Xu et al., 2021).

Although this study clarified the growth-promoting mechanisms of the three optimal strains, limitations still exist: the conditions of pot experiments differ from those of field environments, and the long-term effects of the strains need to be verified in field trials; multi-omics data only reveal the correlation between pathways and gene expression, and the functions of key regulatory factors need to be verified through molecular experiments; the interaction effects between the strains and rhizosphere microorganisms have not been explored in depth. Future research can verify the functions of core genes and metabolites through molecular experiments, and combine microbiomics to analyze the regulatory effects of strains on the rhizosphere microecology.

In conclusion, the three strains of endophytic ascomycetes (Ta121, Cp78, Pe167) screened in this study exhibit significant growth-promoting effects on *F. sinensis* with differentiated core mechanisms. This study enriches the resources of beneficial endophytic fungi in alpine regions and provides theoretical basis and strain support for elucidating the fungus-forage interaction mechanism and developing microbial regulation technologies for forage in the Qinghai-Tibet Plateau.

## 5 Conclusion

In this study, three endophytic ascomycetes strains (Ta121, Cp78, Pe167) were isolated and screened based on their significant growth-promoting effects on *F. sinensis*. Multi-omics analyses revealed that these three strains formed distinct genus-specific growth-promoting mechanisms. *Trichoderma* strains promote plant growth by synthesizing auxins and regulating genes and transcription factors related to plant hormone signal transduction pathways; *Cladosporium* strains achieve growth-promoting effects by secreting steroid hormones and regulating their signaling pathways; the growth-promoting mechanism of *Penicillium* strains is characterized by enhancing carbon cycling processes including plant photosynthesis, glycolysis, and starch and sucrose metabolism.

## Data Availability

RNA-seq data are publicly available in the NCBI database (https://www.ncbi.nlm.nih.gov/) under project number PRJNA1164999.
