# Supplementary material for: Comparative study on the molecular mechanisms of Trichoderma, Cladosporium and Penicillium strains (Ascomycota) in positively regulating Festuca sinensis cv. Qinghai based on multi-omics
Source: Front Microbiol. 2026 Mar 25;17:1788491. doi: 10.3389/fmicb.2026.1788491 (PMC13057412; doi:10.3389/fmicb.2026.1788491)
Supplement: Supplementary file 2 [file Table_2.docx]

Supplementary material

## **Method S1**

1. Untargeted metabolomics analysis of forage roots

A total of 100 mg of tissue samples ground in liquid nitrogen were placed into Eppendorf (EP) tubes, followed by the addition of 500 μL of 80% aqueous methanol solution. The EP tubes containing the samples and reagents were vortexed for 30 s on a vortex oscillator, and then incubated on ice for 5 min. After incubation, the mixtures were centrifuged at 15,000 × g and 4 °C for 20 min. An appropriate volume of the supernatant obtained from the first centrifugation was carefully aspirated and diluted with mass spectrometry-grade water until the methanol concentration in the solution was reduced to 53%. The diluted solution was centrifuged again under the same conditions (15,000 × g, 4 °C, 20 min), and the resulting supernatant was collected and injected into a liquid chromatography-mass spectrometry (LC-MS) system for analysis.

The chromatographic separation was performed on a Hypersil Gold C18 column. The column temperature was maintained at 40 °C, and the flow rate was set at 0.2 mL/min. For the positive ion mode, the mobile phases consisted of Phase A (0.1% formic acid aqueous solution) and Phase B (methanol). For the negative ion mode, the mobile phases were composed of Phase A (5 mM ammonium acetate solution, pH 9.0) and Phase B (methanol). The mass scan range was set at m/z 100–1500. An electrospray ionization (ESI) source was employed with the following parameters: spray voltage 3.5 kV, sheath gas flow rate 35 psi, auxiliary gas flow rate 10 L/min, ion transfer tube temperature 320 °C, S-lens RF level 60, and auxiliary gas heater temperature 350 °C. Both positive and negative ion modes were included in the detection. Data-dependent acquisition (DDA) mode was adopted for MS/MS secondary scanning.

2. Data preprocessing and metabolite identification

The raw data were imported into the CD 3.3 database search software. Initial screening was performed based on retention time and mass-to-charge ratio (m/z), with the peak area calibrated using the first quality control (QC) sample. Subsequently, parameters including a mass deviation of 5 ppm and a signal intensity deviation of 30% were set for peak extraction and quantification. After integrating target ions, molecular formulas were predicted by combining molecular ion peaks and fragment ion peaks, followed by database matching against mzCloud, mzVault and Masslist databases. Blank samples were used to eliminate background ions. The original quantitative results were normalized using the formula: (Raw quantitative value of the metabolite in the sample) / (Total quantitative value of metabolites in the sample / Total quantitative value of metabolites in the QC1 sample) to obtain relative peak areas. Compounds with a coefficient of variation (CV) of relative peak areas > 30% in QC samples were excluded. Finally, the results of metabolite identification and relative quantification were generated.

3. Data statistical analysis

Functional annotation of the identified metabolites was conducted using the KEGG (https://www.genome.jp/kegg/pathway.html), HMDB (https://hmdb.ca/metabolites) and LIPIDMaps (http://www.lipidmaps.org/) databases.

## **Method S2**

1. Total RNA extraction and quality detection from forage roots

Total RNA was extracted from the roots of forage in different treatment groups using the Trizol method, following the manufacturer’s instructions for the Trizol reagent. After extraction, the integrity of the nucleic acid samples was verified by agarose gel electrophoresis. The OD value of the nucleic acids was measured using a NanoDrop spectrophotometer to evaluate nucleic acid purity.

2. Construction of transcriptome library and sequencing

mRNA Capture Beads were removed from the 2–8 °C storage environment and equilibrated to room temperature by standing for 30 min. mRNA purification and fragmentation were performed using an mRNA capture kit following the manufacturer’s protocol. After purification, the size distribution of the mRNA products was analyzed using an Agilent 2100 Bioanalyzer. Upon completion of fragmentation, the samples were placed on a magnetic stand. Once the solution became clear, 17 μL of the supernatant was aspirated into new enzyme-free centrifuge tubes and directly used for first-strand cDNA synthesis.

The reaction system for first-strand cDNA synthesis had a total volume of 25 μL, consisting of 6 μL Strand Specificity Reagent, 2 μL 1st Strand Enzyme Mix, and 17 μL fragmented mRNA. The reaction system was placed in a preheated PCR thermocycler (lid temperature: 105 °C) with the following program: 25 °C for 10 min; 42 °C for 15 min; 70 °C for 15 min; and holding at 4 °C. Immediately after the first-strand reaction, the second-strand cDNA synthesis was carried out. The total volume of the second-strand reaction system was 60 μL, containing 25 μL first-strand cDNA, 30 μL 2nd Strand Buffer (dNTP or dUIP), and 5 μL 2nd Strand Enzyme Master Mix. The reaction system was placed in a preheated PCR thermocycler (lid temperature: 105 °C) with the following program: 16 °C for 30 min; 72 °C for 15 min; and holding at 4 °C.

Subsequently, adapter ligation was performed immediately. The ligation reaction system had a total volume of 100 μL, including 60 μL dA-tailed DNA, 30 μL Ligation Enhancer, 5 μL Novel T4 DNA Ligase, and 5 μL DNA Adapter. The reaction system was incubated in a PCR thermocycler with the program: 20 °C for 15 min; holding at 4 °C. After ligation, the ligation products were purified using the Hieff NGS® DNA Selection Beads kit according to the manufacturer’s instructions.

Library amplification: Amplification reactions were divided into two groups (short adapter and long adapter) based on adapter length. The total volume of each reaction system was 50 μL. The detailed PCR reaction systems and programs are shown in the following table:

| Reagent | Volume | Cycle step | Temperature | Time | Cycles |
| --- | --- | --- | --- | --- | --- |
|  | (μl) |  |  |  |  |
| 2×Super Canace Ⅱ High-Fidelity Mix | 25 µL | Initial Denaturation | 98℃ | 1 min | 1 |
| Universal Primer/i5 Primer | 2.5 µL | Denaturation | 98℃ | 10 s | 12 |
| Index Primer/i7 Primer | 2.5 µL | Annealing | 60℃ | 75 s |  |
| Adapter Ligated DNA | 20 µL | Extension | 72℃ | 30 s |  |
| Total Volume | 50 µL | Final Extension | 72℃ | 5 min | 1 |
|  |  | Hold | 4℃ | Forever | 1 |

| Reagent | Volume | Cycle step | Temperature | Time | Cycles |
| --- | --- | --- | --- | --- | --- |
|  | (μl) |  |  |  |  |
| 2×Super Canace Ⅱ High-Fidelity Mix | 25 µL | Initial Denaturation | 98℃ | 1 min | 1 |
| Primer Mix | 5 µL | Denaturation | 98℃ | 10 s | 12 |
| Adapter Ligated DNA | 20 µL | Annealing | 60℃ | 75 s |  |
| Total Volume | 50 µL | Extension | 72℃ | 30 s |  |
|  |  | Final Extension | 72℃ | 5 min | 1 |
|  |  | Hold | 4℃ | Forever | 1 |

The amplified products were purified using the Hieff NGS® DNA Selection Beads kit following the manufacturer’s protocol. Subsequently, library quality control was performed using the Agilent High Sensitivity DNA Assay Kit, operated in accordance with the kit’s instructions. Libraries that passed quality control were sequenced on the Illumina NovaSeq X Plus platform.

3. Bioinformatics analysis

Raw data generated from the Illumina platform were filtered using fastp software to obtain high-quality clean reads. The filtering criteria were set as follows: (1) removal of reads containing adapter sequences; (2) removal of reads with an N (unknown base) content exceeding 10%; (3) removal of reads composed entirely of adenine (A) bases; (4) removal of low-quality reads (reads where bases with a quality score Q ≤ 20 accounted for more than 50% of the total read length). Mixed assembly of clean reads from all samples was performed using Trinity software to generate transcripts.

Functional annotation of the assembled transcripts was conducted by alignment against multiple databases including CDD, KOG, COG, NR, NT and PFAM using NCBI Blast+. KEGG annotation of the transcripts was obtained via the KAAS server. Coding sequence (CDS) prediction was implemented based on the Blast alignment results combined with TransDecoder software. Valid data of each sample were mapped to the assembled transcripts using Bowtie2, and the mapping statistics were summarized. Redundancy sequence analysis and insert size distribution analysis were performed based on the mapping results using RSeQC. Uniformity distribution inspection and gene coverage statistics were carried out with BEDTools. Potential single nucleotide polymorphism (SNP) loci were identified from the mapping results using BCFtools for subsequent SNP analysis. Simple sequence repeat (SSR) analysis was performed based on the assembled transcript sequences using MISA software. Gene expression levels were quantified using Salmon software. Differential gene expression analysis was conducted with DESeq2, and visualization was performed for the results of differential expression analysis.


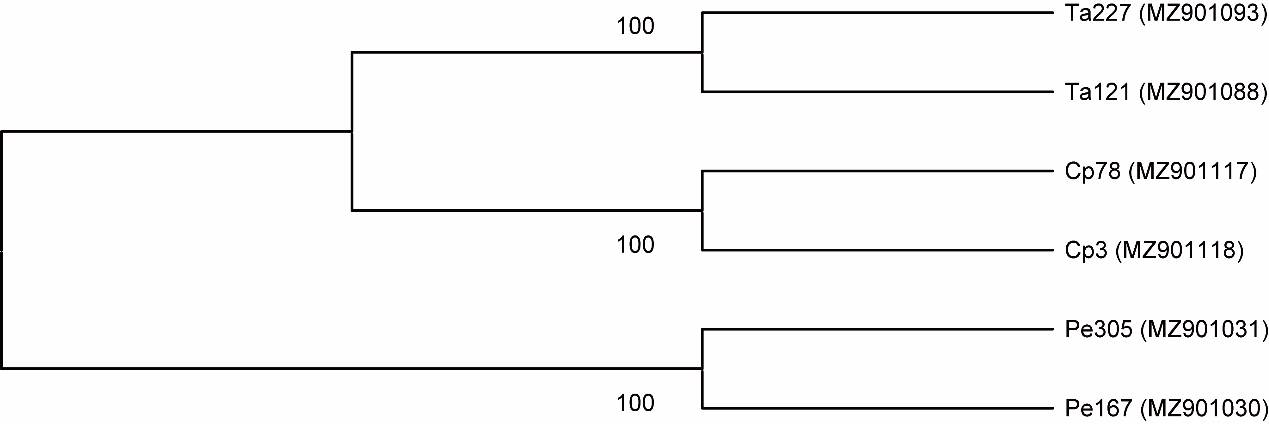


Fig S1. Phylogenetic tree of six endophytic Ascomycete strains based on ITS sequences. The tree was constructed using the neighbor-joining method in MEGA 11. Bootstrap values (1000 replicates) are shown at the nodes. The GenBank accession numbers are provided in parentheses.


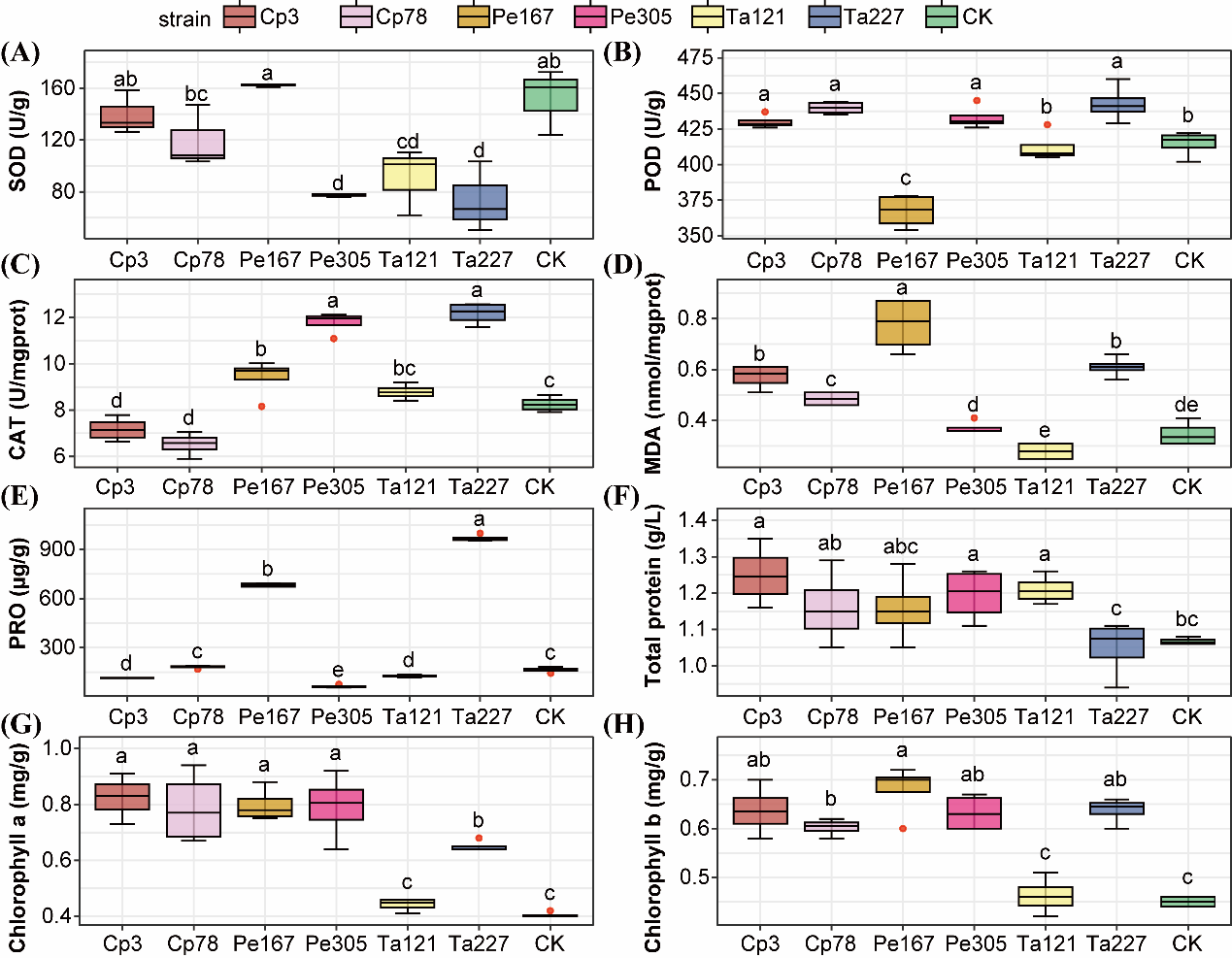


Fig S2. Effects of different strains on biochemical substance contents in leaves of *Festuca sinensis* cv. Qinghai. (A) superoxide dismutase (SOD) activity; (B) peroxidase (POD) activity; (C) catalase (CAT) activity; (D) malondialdehyde (MDA) content; (E) proline (PRO) content; (F) Total protein content; (G) chlorophyll a content; (H) chlorophyll b content. Statistical analysis was performed using Student's t-test. Different lowercase letters above the boxplots indicate significant differences (p < 0.05), n = 4.


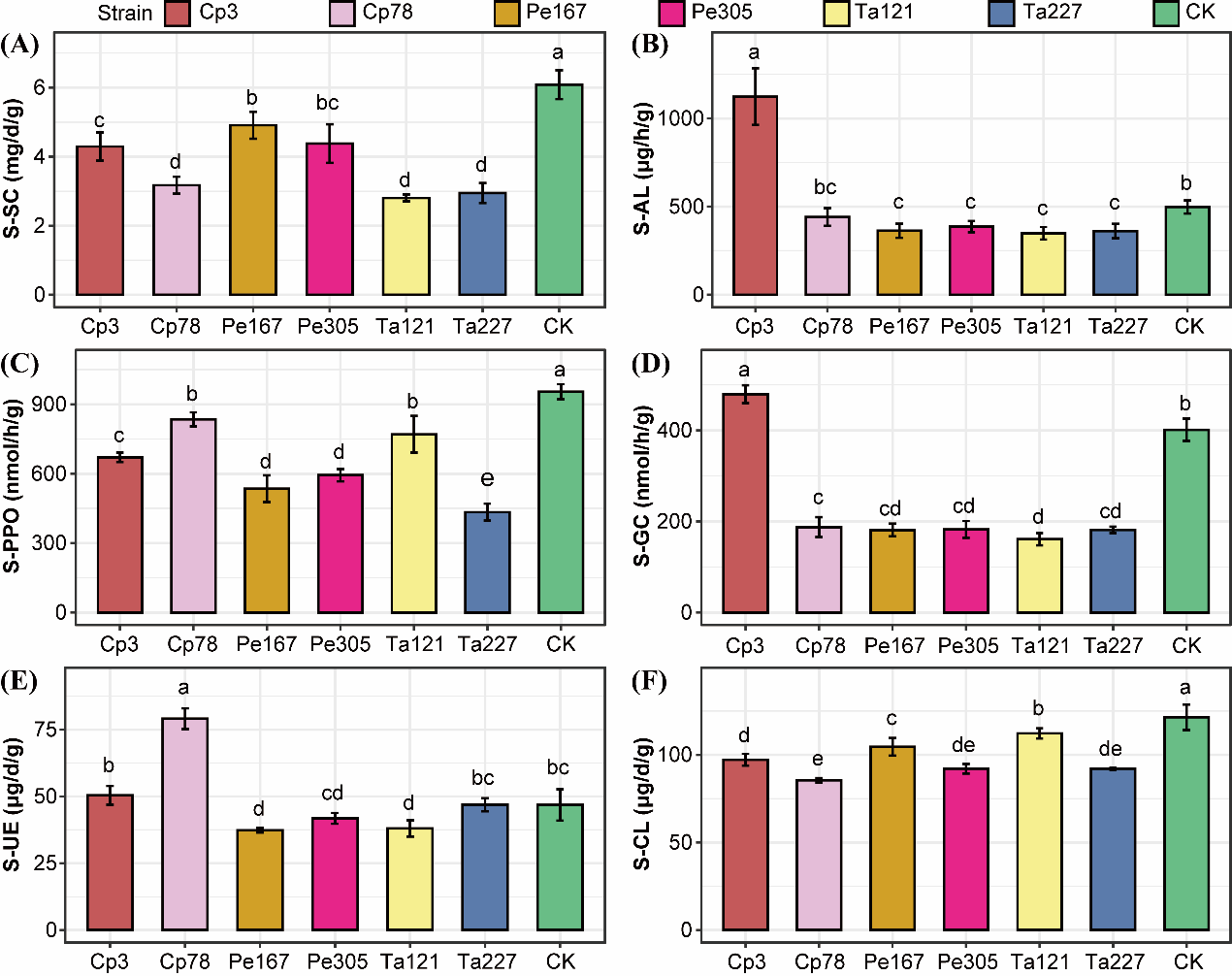


Fig S3. Effects of different strains on enzyme activities in rhizosphere soil of *Festuca sinensis* cv. Qinghai. (A) invertase (S-SC); (B) amylase (S-AL)；(C) polyphenol oxidase (S-PPO); (D) glucosidase (S-GC); (E) urease (S-UE); (F) cellulase (S-CL). Statistical analysis was performed using Student's t-test. Different lowercase letters above the columns indicate significant differences (p < 0.05), n = 4.


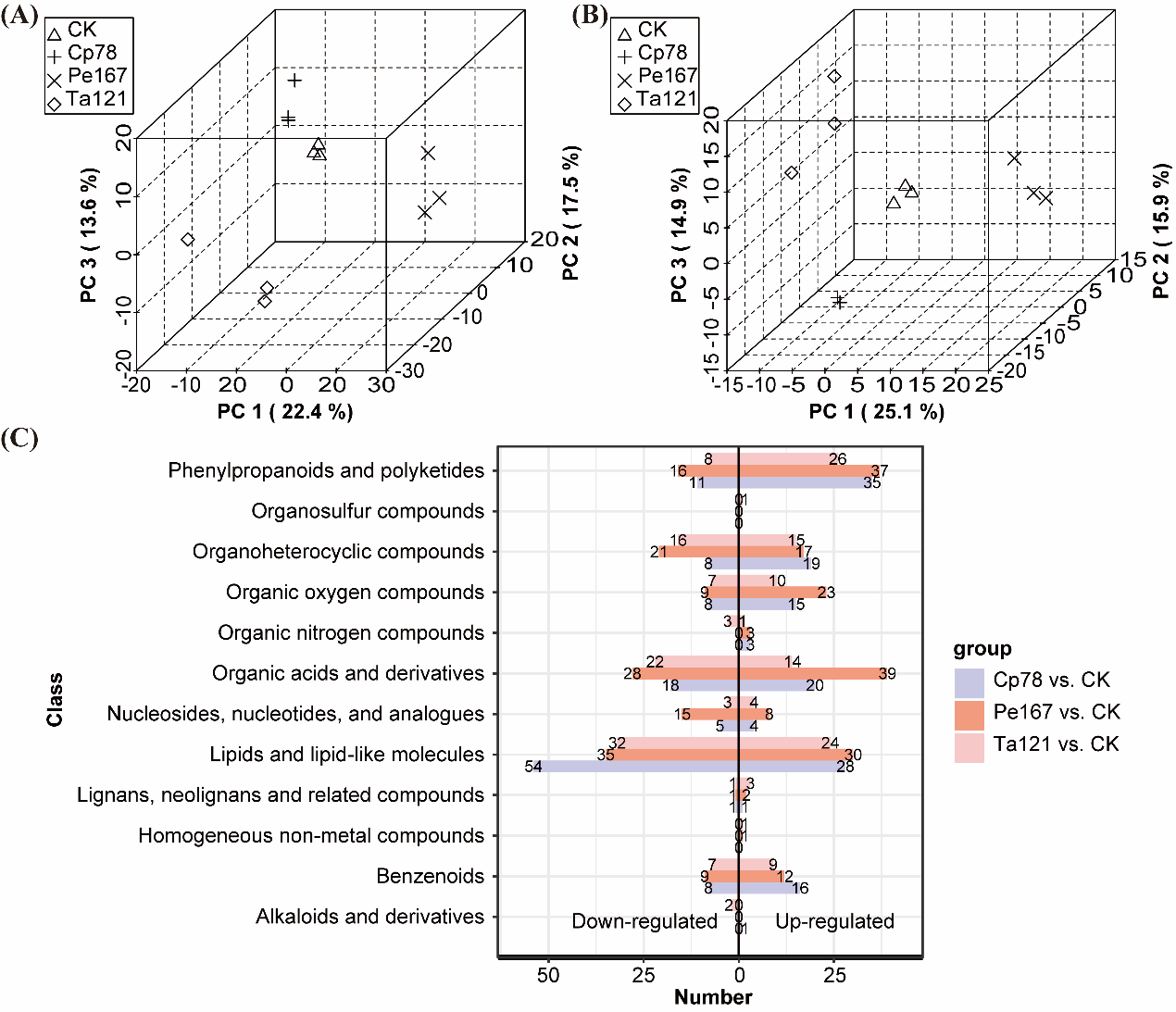


Fig S4. Intergroup principal component analysis (PCA) score plots in positive (A) and negative ion modes (B); (C) functional classification plot of significantly differential metabolites (SDMs) between treatment and control groups.


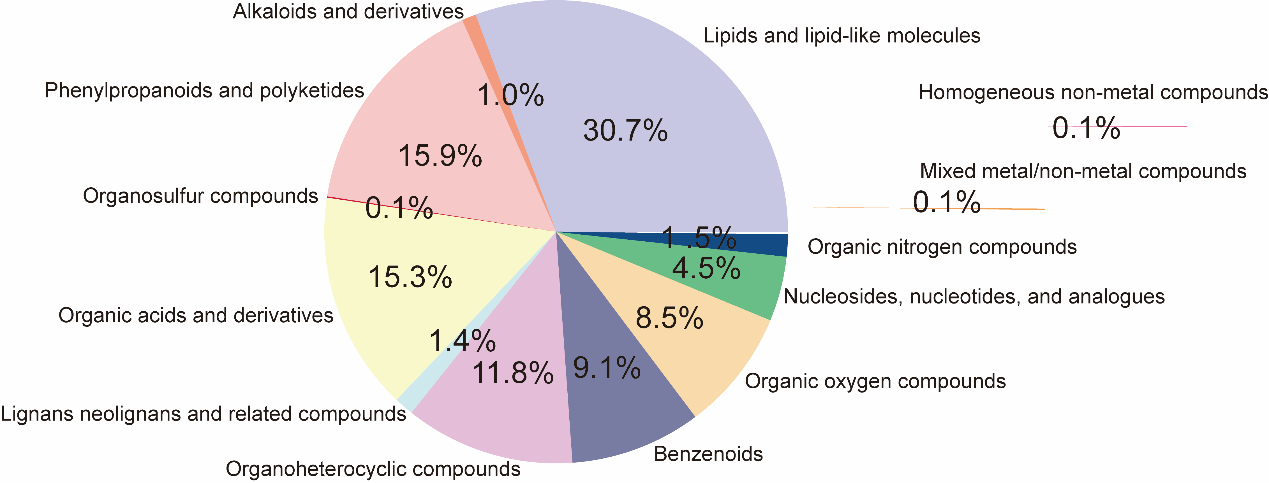


Fig S5. Biological functional classification and proportion of metabolites in forage roots.


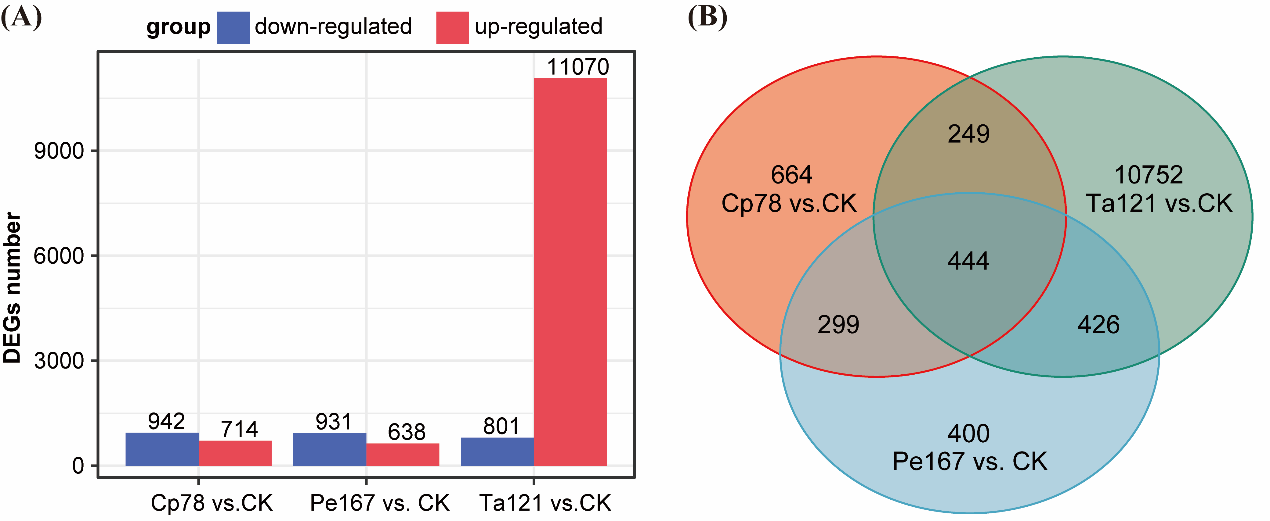


Fig S6. Screening of differentially expressed genes (DEGs) between different treatment and control groups. (A) Bar chart of DEG numbers among different comparison groups; (B) venn diagram of DEGs among different comparison groups.


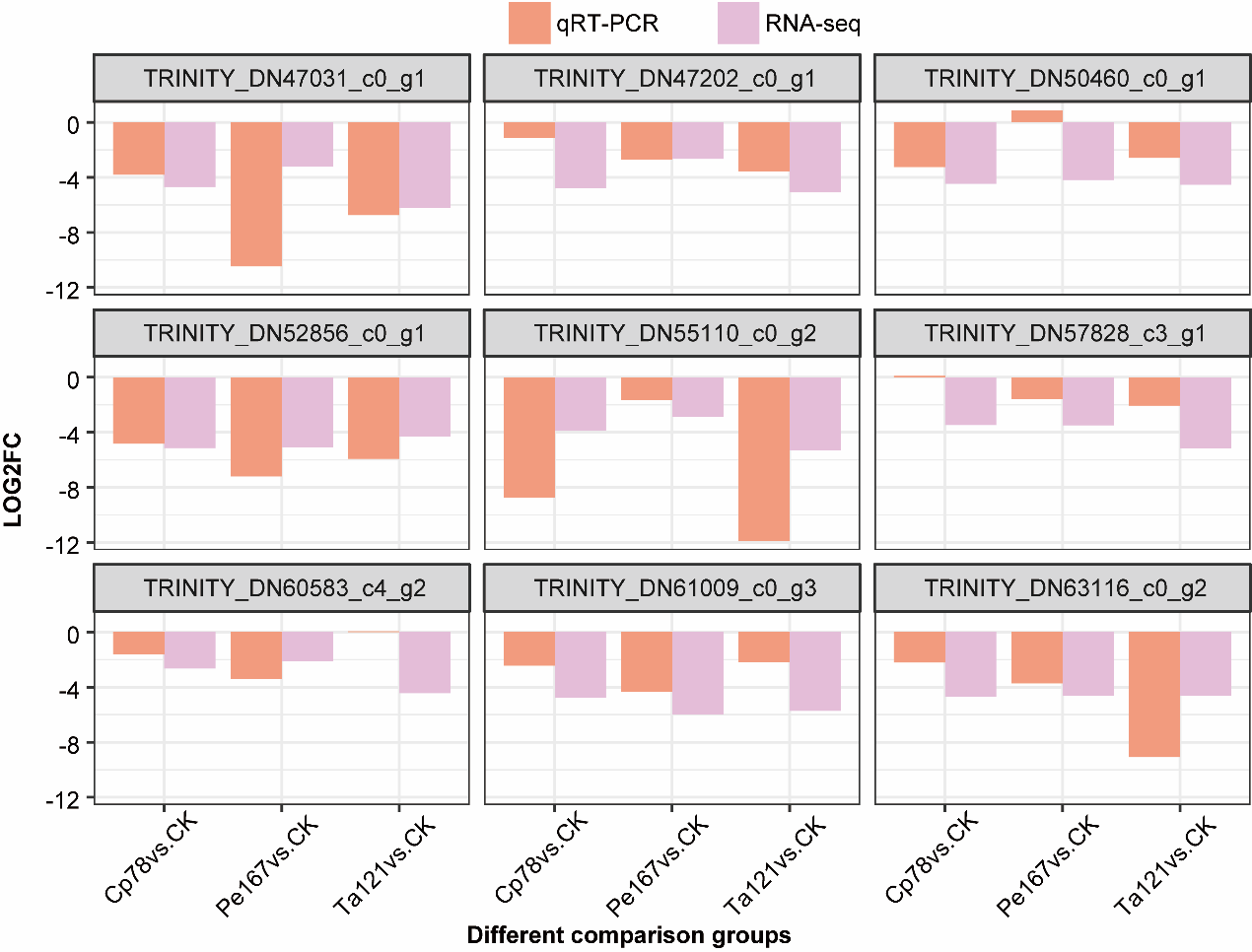


Fig S7. RT-qPCR validation of RNA-seq data for nine differentially expressed genes. Blue bars represent RNA-seq data, and red bars represent quantitative real-time PCR (RT-qPCR) data.


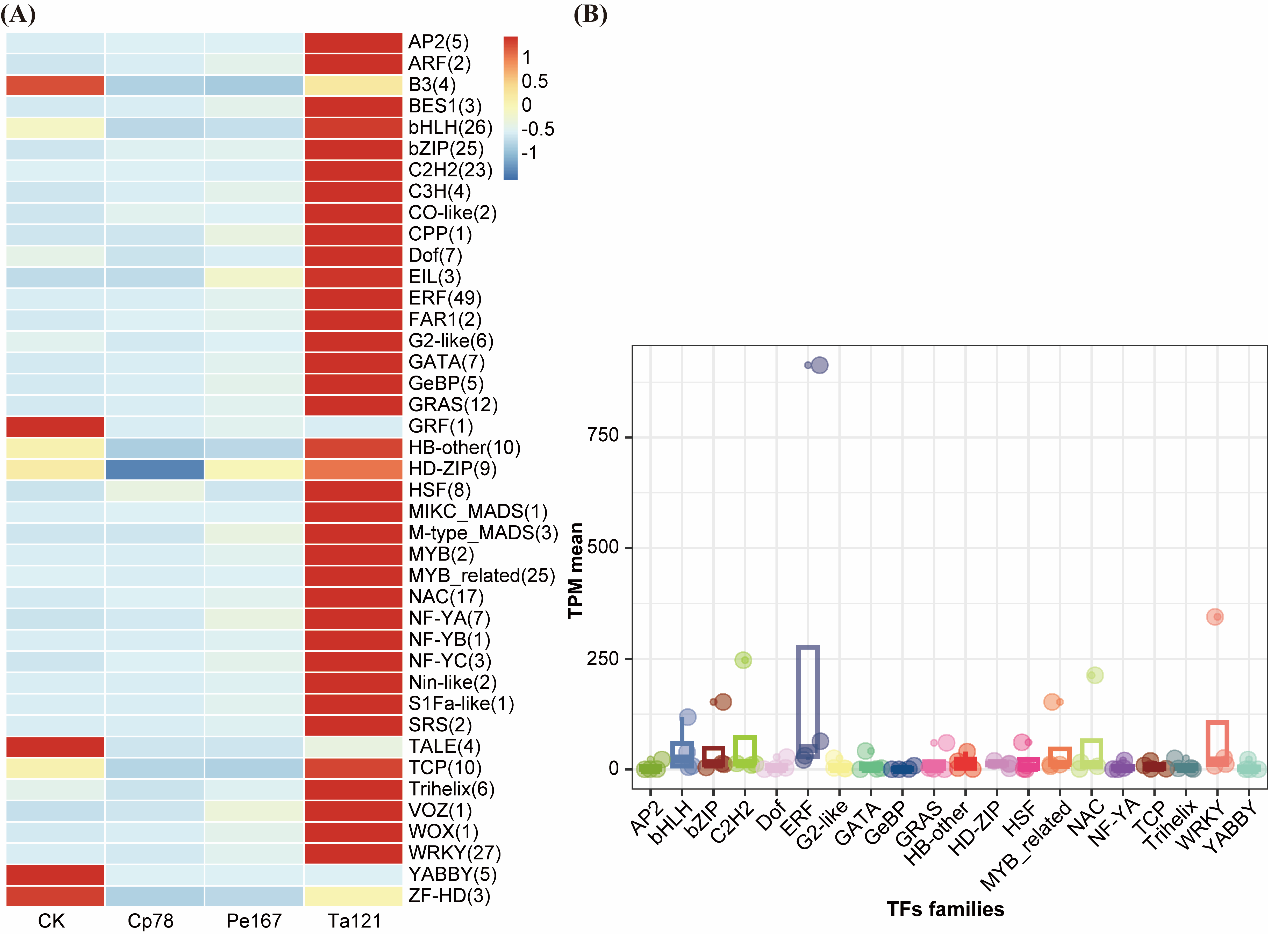


Fig S8. Expression patterns of transcription factor families. (A) Heatmap of expression levels of different transcription factor families under different microbial treatments (color indicates the normalized total TPM value of all members in the corresponding transcription factor family; numbers in parentheses represent the total number of transcription factors in the family); (B) Boxplot of different transcription factor families under four treatment conditions (ordinate represents the mean TPM value of the corresponding transcription factor family across the four treatments).


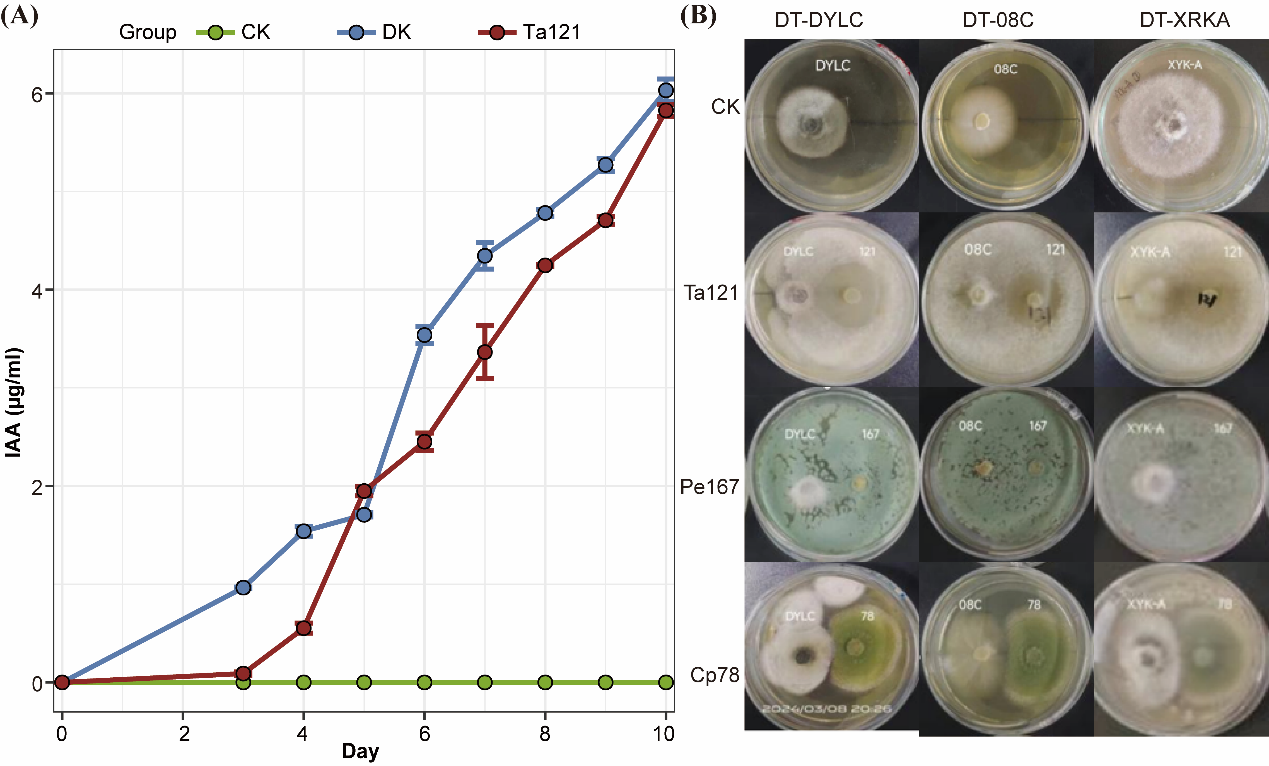


Fig. S9. Detection of indole-3-acetic acid (IAA) production capacity and plant pathogen antagonistic activity of the strains. (A) Dynamic changes in IAA production of *Trichoderma* strain Ta121 and the high-efficiency plant growth-promoting fungal strain DK over different culture periods; (B) Antagonistic effects of strains Ta121, Pe167 and Cp78 against plant pathogens. *Fusarium paeoniae* DT-08C, *Alternaria alternata* strains DT-DYLC and DT-XRKA were used as the tested plant pathogenic fungi.
